# TET1 Inhibition Promotes Therapeutic Sensitivity in TP53‐Mutant GBM by Influencing Genome Fragility and Altering TAMs Biology

**DOI:** 10.1002/advs.76517

**Published:** 2026-07-11

**Authors:** Zhuonan Pu, Jinqiu Liu, Yuxuan Deng, Shuyu Hao, Xiaoli Zhang, Mingxu Yang, Chao Guo, Chao Du, Yingdan Chen, Tai Sun, Nan Ji, Zhengping Zhuang, Jie Feng

**Affiliations:** ^1^ Beijing Neurosurgical Institute Capital Medical University Beijing China; ^2^ Department of Neurosurgery Beijing Tiantan Hospital Capital Medical University Beijing China; ^3^ Neuro‐Oncology Branch Center for Cancer Research National Cancer Institute National Institutes of Health Bethesda MD USA

**Keywords:** epigenetic change, Glioblastoma, TET1, therapeutic resistance, TP53‐mutant

## Abstract

TP53 mutations (TP53mut) are associated with therapeutic resistance in glioblastoma (GBM) patients, yet the underlying mechanisms remain incompletely understood. Here, we identified an association between reduced P53 function and increased expression of the epigenetic regulator TET1 in GBM models. In TP53mut GBM cells, TET1 knockdown influenced genome fragility, including DNA damage, senescence, and telomere shortening. Specifically, our findings are consistent with a model in which TET1 binds to the ROS1 promoter and may help maintain ROS1 expression, likely by keeping the promoter in a hypomethylated state, along with downstream ERK phosphorylation. Conversely, inhibiting TET1 correlates with reduced ROS1 expression, attenuation of ERK signaling, and increased genome fragility. Furthermore, TET1 depletion in tumor cells was associated with altered tumor‐associated macrophages biology both in vitro and in vivo, including increased infiltration, differentiation, M1‐like polarization, and phagocytic capacity. Notably, combining the TET1 inhibitor Bobcat339 with cisplatin synergistically inhibited TP53mut GBM growth in vitro and in vivo, improving survival without significant toxicity. Our findings suggest that TET1 may serve as a potential mediator of therapy resistance and a promising therapeutic target in TP53mut GBM.

## Graphical abstract

In TP53mut GBM cells, reduced P53 function is associated with increased TET1 expression. Genetic or pharmacological inhibition of TET1 correlates with genome fragility, including DNA damage, cellular senescence, telomere shortening, and reactive oxygen species accumulation, which may contribute to increased efficacy of antitumor therapy. TET1 inhibition is associated with ROS1 promoter hypermethylation, reduced ROS1 expression, and subsequent suppression of ERK phosphorylation. Moreover, TET1 inhibition promotes TAMs differentiation, M1‐type polarization, infiltration, and phagocytosis, which may suggest a role in TAMs‐targeted treatment. In multiple GBM cell lines in vitro and in orthotopic xenografic models in vivo, TET1 inhibition synergizes with cisplatin to suppress TP53mut GBM tumor growth, which may represent a promising therapeutic strategy.

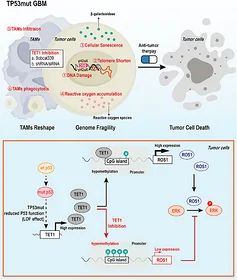

## 1 Introduction

Glioblastoma (GBM) is the most common and aggressive primary intracranial malignant tumor in adults, and patients with GBM have a median survival of less than 2 years [1]. Despite numerous treatment strategies, the therapeutic efficacy remains limited, and survival rates have not yet improved. GBM has a high frequency of TP53 mutation (TP53mut), which is reported in more than 50% of cases and is strongly associated with poor overall survival [2]. Mutant P53 not only exhibits loss of function (LOF) owing to the lack of the wild‐type protein's tumor suppressor function and the failure to activate canonical target genes, but also acquires gain‐of‐function (GOF) through the activation of noncanonical target genes [3]. Both LOF and GOF in TP53mut promote tumor progression.

In addition to promoting GBM growth, TP53mut confers resistance to diverse therapeutic modalities. These include cytotoxic agents directed at tumor cells, as well as therapies targeting the tumor microenvironment (TME), such as antiangiogenic agents, modulators of tumor‐associated macrophages (TAMs), and immune checkpoint inhibitors [4, 5]. Although numerous clinical trials have been conducted for TP53mut GBM, focusing on both tumor cells and their TME, most have encountered limited success because of rapid adaptation and acquired resistance. Thus, investigating how TP53mut may contribute to therapeutic resistance in GBM is essential for improving treatment outcomes and patient survival. Elucidating the underlying mechanisms may reveal novel therapeutic targets and increase treatment sensitivity.

Emerging evidence implicates genome fragility restriction as a key facilitator of TP53mut‐mediated resistance. This fragility is characterized by persistent DNA damage, induction of cellular senescence [6], structural chromosomal alterations (e.g., telomeric and centromeric abnormalities) [7], and accumulation of reactive oxygen species [8]. Notably, inducing genome fragility can synergistically increase the efficacy of various anticancer therapies, including chemotherapy, radiotherapy, and immunotherapy [9, 10, 11]. In fact, TP53mut have been shown to mitigate genome fragility [12, 13, 14, 15]. However, in TP53mut GBM, the critical molecules that may regulate genome fragility remain unknown, and effective intervention strategies targeting this process are lacking.

Research has demonstrated that epigenetic dysregulation contributes to genome fragility and plays a critical role in therapeutic resistance mediated by TP53mut. Tet methylcytosine dioxygenase 1 (TET1) acts as a master epigenetic regulator by catalyzing the oxidation of 5‐methylcytosine (5mC) to 5‐hydroxymethylcytosine (5hmC), thereby driving DNA demethylation and gene regulation [16, 17]. Due to its potential to induce aberrant DNA demethylation, TET1 downregulation or dysfunction correlates with profound epigenetic alterations. TET1 expression can be suppressed by miRNAs, regulatory proteins, or promoter methylation; for instance, wild‐type P53 has been shown to bind to the TET1 promoter and suppress its transcription in lung cancer cells [18]. Moreover, TET1 dysregulation contributes to genome fragility in tumors, potentially affecting the DNA damage response (DDR), key signaling pathways, and telomere integrity [19, 20]. In GBM, upregulated TET1 expression has been reported to impair DNA repair mechanisms, thereby reducing the response to ionizing radiation [21]. The role of TET1‐mediated epigenetic reprogramming and genome fragility in facilitating therapeutic resistance in TP53mut tumors is currently unknown, and the underlying mechanisms are unexplored. To address this challenge, we explored therapeutic strategies that combine conventional chemotherapeutics with inhibitors targeting TET1‐associated pathways as a potential approach to overcome resistance in these tumors.

## 2 Results

### 2.1 Elevated TET1 Expression in GBM Harboring TP53mut

To elucidate the molecular basis of therapeutic resistance in GBM with TP53mut and identify novel therapeutic targets, we conducted differential gene expression profiling in TP53mut and TP53wt samples obtained from the TCGA database. Sex, age, and therapy were not considered biological variables (Figure S1A). Furthermore, we conducted GSEA on the basis of gene ontology terms (GO 0006304) and observed significantly different enrichment scores for DNA modifications. To explore potential DEGs involved in DNA modification between TP53mut and TP53wt samples, we conducted Limma analysis to identify DEGs with a p value <0.05 and |fold change| >1.5, and among those identified, only TET1 was associated with DNA modification (Figure 1A,B). To assess clinical relevance, we performed immunohistochemical (IHC) staining to quantitatively evaluate TET1 expression in IDHwt GBM tissues, with staining intensity scored and stratified by TP53 status. The results showed that TET1 expression levels were significantly higher in TP53mut GBM samples (Figure S1B,C) than in TP53wt samples (Figure S1D,E).

**FIGURE 1:**
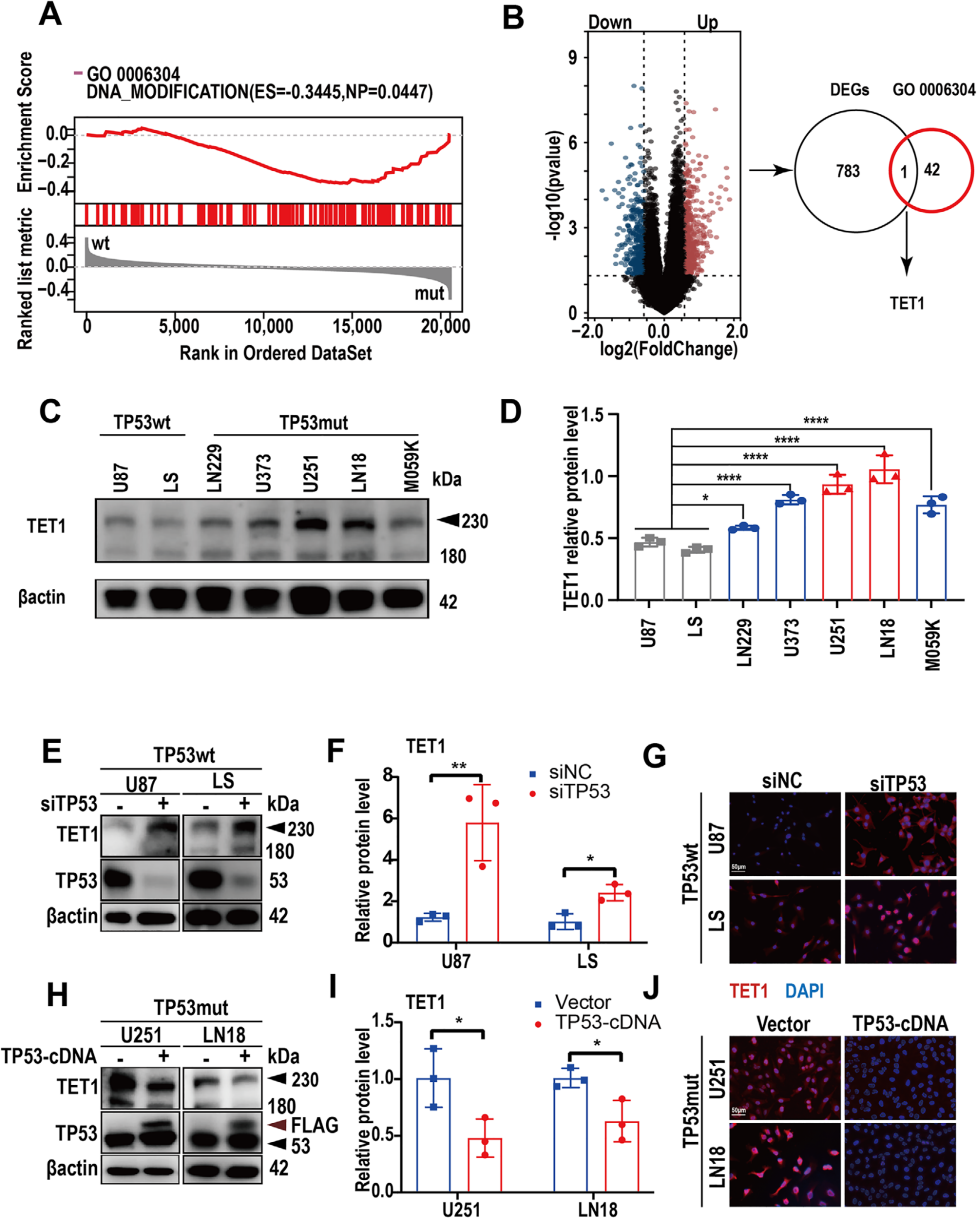
Reduced P53 function correlates with the TET1 expression in GBM. (A) GSEA of DNA modifications (GO0006304) in IDHwt GBM samples with TP53wt and TP53mut from the TCGA cohort. (B) The filtering process showing the overlap of the gene sets containing DEGs related to DNA modification between TP53wt and TP53mut GBM samples. (C, D) Western blot analysis and quantification of TET1 expression in different GBM cell lines. (E, F) Western blot analysis and quantification of TET1 expression in TP53wt U87 and LS GBM cell lines transfected with siTP53 or siNC. (G) Immunofluorescence analysis of TET1 expression in U87 and LS cells transfected with siTP53 or siNC. (H, I) Western blot analysis and quantification of TET1 expression in TP53mut U251 and LN18 cells expressing TP53‐cDNA or empty vector. (J) Immunofluorescence staining of TET1 in TP53mut U251 and LN18 cells expressing TP53‐cDNA or empty vector. TET1, red; DAPI, blue. *n* = 3; data are presented as the means ± SDs (D, F, and I). **P < 0.01, ****P < 0.0001.

Moreover, our results revealed greater expression of TET1 in TP53mut GBM cell lines (LN229, U373, U251, LN18, and M059K) than in TP53wt cell lines (U87 and LS). The highest TET1 expression was observed in U251 and LN18 cells, which have R273H and C238S mutations, respectively (Figure 1C,D and Figure S2A,B); these cells were selected for further experiments.

To observe the effect of TP53 on TET1 expression, we transfected siRNA to interfere with TP53 expression in TP53wt cell lines because the main consequence of TP53mut is LOF. As expected, the RNA and protein expression levels of TET1 were significantly greater in TP53wt cells transfected with TP53‐siRNA than in those transfected with siNC (Figure 1E,F and Figure S2C). Conversely, we transfected TP53‐cDNA‐FLAG into TP53mut cell lines and detected decreased TET1 RNA and protein expression levels compared with those in vector‐treated cells (Figure 1H,I and Figure S2D). We further performed immunofluorescence (IF) to examine TET1 subcellular localization under TP53 knockdown and TP53 overexpression conditions. Our results showed that upon TP53 depletion, TET1 expression markedly increased, and this factor remained predominantly localized to the nucleus (Figure 1G). In contrast, TP53 overexpression reduced nuclear TET1 expression (Figure 1J). These findings revealed that the changes in TET1 levels detected by Western blotting reflected alterations in nuclear expression, which is consistent with its function. Overall, the results suggest that in TP53wt cells, siRNA‐mediated inhibition of TP53 correlates with the upregulation of TET1 expression, whereas TP53 overexpression in TP53mut cells is associated with the reduction of TET1 expression.

### 2.2 Knocking Down TET1 Expression Promotes Genome Fragility in TP53mut GBM

To investigate the effect of TET1 in GBM with TP53mut on DNA damage, cellular senescence, telomere shortening, and oxidative stress, we performed a TET1 knockdown model by treating TP53mut GBM cells with TET1‐shRNA or TET1‐siRNA (Figure S3A,B). First, DNA damage was quantified via IF by determining the average number of γ‐H2AX foci per cell. As expected, the introduction of either TET1‐shRNA or TET1‐siRNA into TP53mut cells showed a significant increase in γ‐H2AX levels compared with those in their respective controls (shNC or siNC). (Figure 2A,B and Figure S3C). Second, the activity of senescence‐associated beta galactosidase (SA‐β‐Gal), a well‐established marker of cellular senescence, was quantified by counting SA‐β‐Gal‐positive cells. TET1 knockdown (with shTET1 or siTET1) markedly elevated SA‐β‐Gal activity in TP53mut cells compared with that in control cells (shNC/siNC), suggesting the induction of cellular senescence (Figure 2C,D). Third, we performed telomere length analysis by quantitative fluorescence in situ hybridization (Q‐FISH) and quantitative PCR (qPCR). Compared with controls (shNC or siNC), TET1 knockdown (shTET1 or siTET1) significantly shortened telomeres in TP53mut GBM cell lines, as consistently shown by a reduction in telomere fluorescence intensity (Q‐FISH) and presented by qPCR (Figure 2E,F and Figure S3D,E). Next, to assess oxidative stress after TET1 knockdown, we measured the reactive oxygen species levels in the TP53mut cells and detected a significant increase in fluorescence intensity in the shTET1‐transfected cells compared with those in the shNC control cells (Figure S4).

**FIGURE 2:**
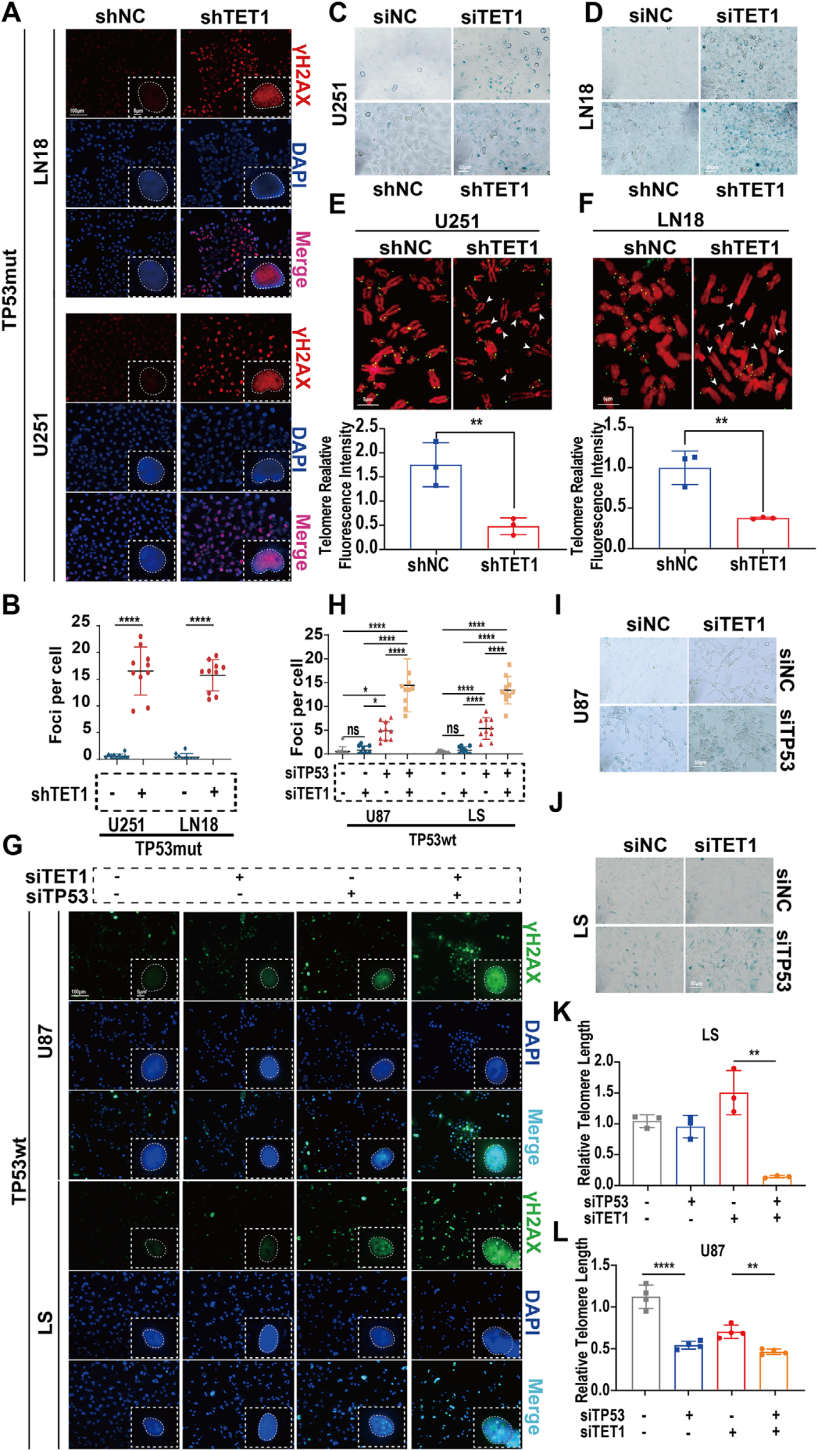
TET1 knockdown contributes to DNA damage, cellular senescence, and telomere shortening. (A, B) Immunofluorescence staining of γ‐H2AX foci in U251 and LN18 cells transfected with shTET1 or shNC. Nuclei were counterstained with DAPI. Foci were counted in at least 20 cells per experiment (*n* = 4). (B). γ‐H2AX, red; DAPI, blue. (C, D) SA‐β‐Gal staining of U251 and LN18 cells transfected with shTET1/siTET1 or shNC/siNC, respectively. (E, F) Telomere intensity in U251 and LN18 cells transfected with shTET1 or shNC, as analyzed by Q‐FISH, and quantification (*n* = 3). Telomeric DNA probe, green; DAPI, red. (G, H) Immunofluorescence staining and quantification of γ‐H2AX foci in U87 and LS cells transfected with siTET1, siTP53, siTET1 and siTP53 or siNC (*n* = 4). γ‐H2AX, green; DAPI, blue. (I, J) SA‐β‐Gal staining of U87 and LS cells transfected with siTET1, siTP53, siTET1 & siTP53, or siNC. (K, L) Quantitative telomere length analysis by qPCR in U87 and LS cells expressing siTET1, siTP53, siTET1 & siTP53, or siNC (*n* = 3). Data are presented as the means ± SDs (B, E, F, H, K, and L). *P < 0.05, **P < 0.01, ****P < 0.0001.

Additionally, we evaluated whether the phenotypes affected by TET1 knockdown were reversible by the DNA hypomethylating agent decitabine (Dec). Using a validated concentration (5 µm) [22], we found that after 96 h of treatment with Dec, the DNA damage (γ‐H2AX) and senescence (SA‐β‐Gal activity) phenotypes in shTET1 TP53mut GBM cells were significantly reversed, but the increase in reactive oxygen species levels was not (Figure S4).

### 2.3 Knockdown of Wild‐Type P53 Function Sensitizes Cells to Genome Fragility Modulated by TET1 Knockdown

To investigate whether the DNA damage, cellular senescence, and telomere shortening influenced by TET1 knockdown depend upon reduced wild‐type P53 function (i.e. TP53 LOF), we introduced TP53 siRNA into TP53wt cells (U87 and LS). The results identified that TET1 knockdown alone (siTET1) did not alter γ‐H2AX levels, whereas TP53 depletion (siTP53) alone increased DNA damage, as expected. Notably, knockdown of both TET1 and TP53 (siTP53 & siTET1) exhibited a significant increase in γ‐H2AX levels, exceeding those observed with the knockdown of each factor individually (siTET1 or siTP53) or that in the control (siNC) (Figure 2G,H).

Consistent with the DNA damage results, TET1 knockdown alone (siTET1) did not significantly alter SA‐β‐Gal activity in TP53wt GBM cells. TP53 depletion (siTP53) alone correlated with senescence, the combined knockdown of TET1 and TP53 significantly increased SA‐β‐Gal activity compared with that in all the other groups (siTET1, siTP53, and siNC) (Figure 2I,J). While TET1 knockdown (siTET1) alone presented no significant change in either U87 or LS cells, TP53 knockdown (siTP53) shortened telomeres in U87 cells but not in LS cells. In contrast, compared with siTET1 alone, the combined knockdown of TET1 and TP53 significantly shortened telomeres in both cell lines (Figure 2K,L).

In conclusion, these findings suggest that the coexistence of TP53 knockdown and the overexpressed TET1 inhibition exhibits a synergistic effect to promote hallmarks of genome fragility, including DNA damage, cellular senescence, and telomere shortening.

### 2.4 TET1 Knockdown Is Associated with Altered TAMs Biology in TP53mut GBM

Given the immunomodulatory role of TET1, we hypothesized that knocking down TET1 in TP53mut GBM cells would reprogram the biology of TAMs, thereby influencing therapeutic resistance. Our data suggest that TET1 knockdown in TP53mut GBM cells affects the differentiation, polarization, migration, and infiltration of TAMs both in vitro and in vivo.

First, we assessed the impact of TET1 knockdown on TAMs differentiation and polarization in vitro by coculturing U937 cells with conditioned medium (CM) from TP53mut GBM cells transfected with shTET1 or shNC. Flow cytometry revealed that compared with coculture with CM from shNC cells, coculture with CM from shTET1 cells promoted a significantly greater proportion of CD68^+^ cells (Figure 3A and Figures S5,S6B). CM from shTET1 cells produced a markedly greater proportion of M1‑type TAMs (CD68^+^CD86^+^) relative to the number induced upon coculture with CM from shNC cells, but the proportion of M2‐type TAMs (CD68^+^CD206^+^) did not significantly differ (Figure S6A–F). Notably, CM from shTET1 cells displayed a significantly higher M1/M2 TAMs ratio, indicating that TET1 knockdown in TP53mut GBM cells promotes TAMs differentiation and their polarization into M1‐like TAMs in vitro (Figure S6G). Next, to assess TAMs migration, we conducted a Transwell assay. We found that compared with CM from shNC control cells, CM from shTET1‐treated TP53mut GBM cells produced a significant increase in the migration of PMA‐differentiated U937 macrophages (Figure 3B,C).

**FIGURE 3:**
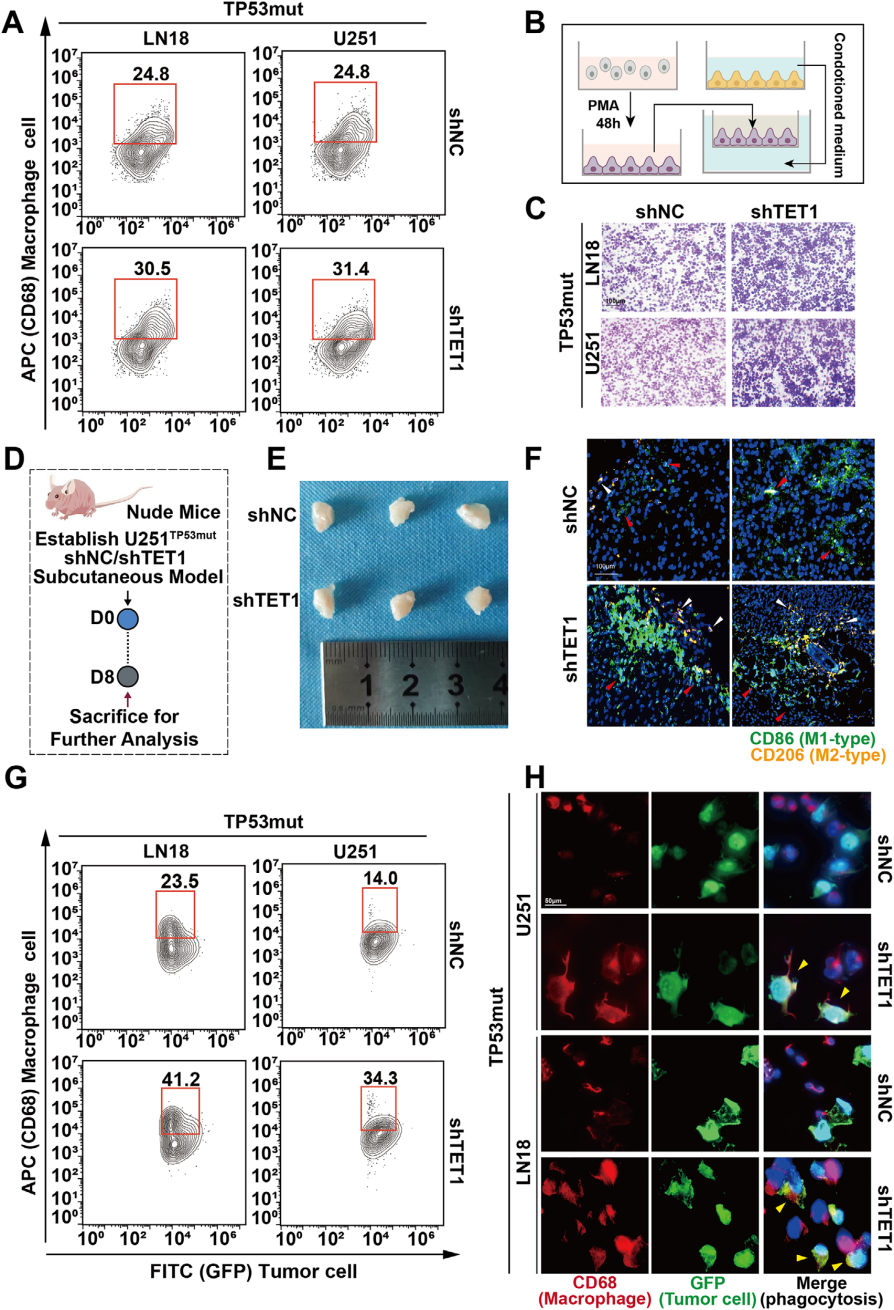
TET1 influences the immunoregulatory function of TAMs. (A) Flow cytometry analysis of the proportion of CD68^+^ TAMs among U937 cells cultured with CM from U251 and LN18 cells transfected with shTET1 or shNC. (B, C) Transwell migration assay results of TAMs cultured in CM from U251 and LN18 cells transfected with shTET1 or shNC. (D) Schematic illustration of the in vivo study timeline. (E) GBM tumor volumes in mice injected with U251 cells transfected with shNC or shTET1. (F) Immunofluorescence staining images of CD86 and CD206 in tumor sections from mice injected with U251 cells transfected with shNC or shTET1. CD86, green; CD206, yellow; DAPI, blue; red arrows, M1‐type TAMs; white arrows, M2‐type TAMs. (G) Flow cytometry analysis of CD68^+^GFP^+^ double‐positive TAMs. (H) Immunofluorescence staining images of CD68+GFP+ double‐positive TAMs. GFP, green; CD68, red; DAPI, blue; yellow arrows, TAMs phagocytosis.

Furthermore, we established a subcutaneous xenograft model by implanting U251 cells transfected with shNC or shTET1 that exhibited stable expression of the related factor into nude mice, which have functional macrophages (Figure 3D). The mice were randomly assigned to one of two groups: one group was injected with shNC‐transduced cells, and the other group was injected with shTET1‐transduced cells. The tumors were harvested and measured at eight days after inoculation. As expected, no significant difference in tumor volume was observed between the two groups (Figure 3E). To assess TAMs infiltration in situ, we performed IF staining for CD86 and CD206, which are well‐established markers of M1‐ and M2‐type TAMs, respectively, and quantified the staining intensity of CD86^+^ and CD206^+^ cells. Compared with those in the shNC group, the intensity of CD86^+^ cells (M1‐type TAMs) in the shTET1 group was significantly greater; additionally, while the intensity of CD206^+^ cells (M2‐type TAMs) in the shTET1 group was greater but the difference was not significant (Figure 3F and Figure S7A–C). Collectively, these results suggest that TET1 knockdown in TP53mut GBM cells promotes TAMs infiltration and polarization both in vitro and in vivo.

Additionally, to investigate whether tumor‐intrinsic TET1 expression influences TAMs phagocytic activity, we cocultured U937‐derived macrophages with GFP‐labeled TP53mut GBM cells transduced with shNC or shTET1. After 48 h of coculture, phagocytosis was assessed by quantifying the number of CD68^+^GFP^+^ double‐positive TAMs using flow cytometry (Figure S5). The results revealed a significantly greater proportion of CD68^+^GFP^+^ cells in the shTET1 group than in the shNC group (Figure 3G). These findings were validated by IF staining, which similarly revealed increased numbers of CD68^+^GFP^+^ TAMs in the shTET1‐treated group (Figure 3H). Together, these data show that the knockdown of TET1 in TP53mut GBM cells may alter TAMs biology, including their differentiation, polarization, migration, infiltration, and phagocytosis capacity.

### 2.5 TET1 Binds to the ROS1 Promoter and Is Associated with CpG Island Methylation Regulation in TP53mut GBM

Collectively, our data observe that the reduced function of P53 relates to the upregulation of TET1 expression in TP53mut GBM. Therefore, we next aimed to investigate how TET1 modulates genome fragility in TP53‐depleted cells. Analysis of key enzymes in major DNA repair pathways in TP53mut GBM cells presented no significant differences after treatment with shNC or shTET1, suggesting that additional factors may contribute to the genome fragility influenced by TET1 knockdown (Figure S8A,B). First, we performed ChIP‐seq to profile the genome‐wide binding sites in TP53mut and TP53wt cells overexpressing TET1 (LvTET1), and *p* value of <0.05 was set for ChIP‐seq peak calling. Genome‐wide mapping of TET1 binding sites revealed a greater number of peaks in LvTET1 TP53mut GBM cells than in LvTET1 TP53wt cells (Figure 4A and Figure S9A–C). Notably, overrepresentation analysis of peak‐associated genes indicated significant enrichment of signal transduction pathways in both genetic contexts (Figure S10A,B).

**FIGURE 4:**
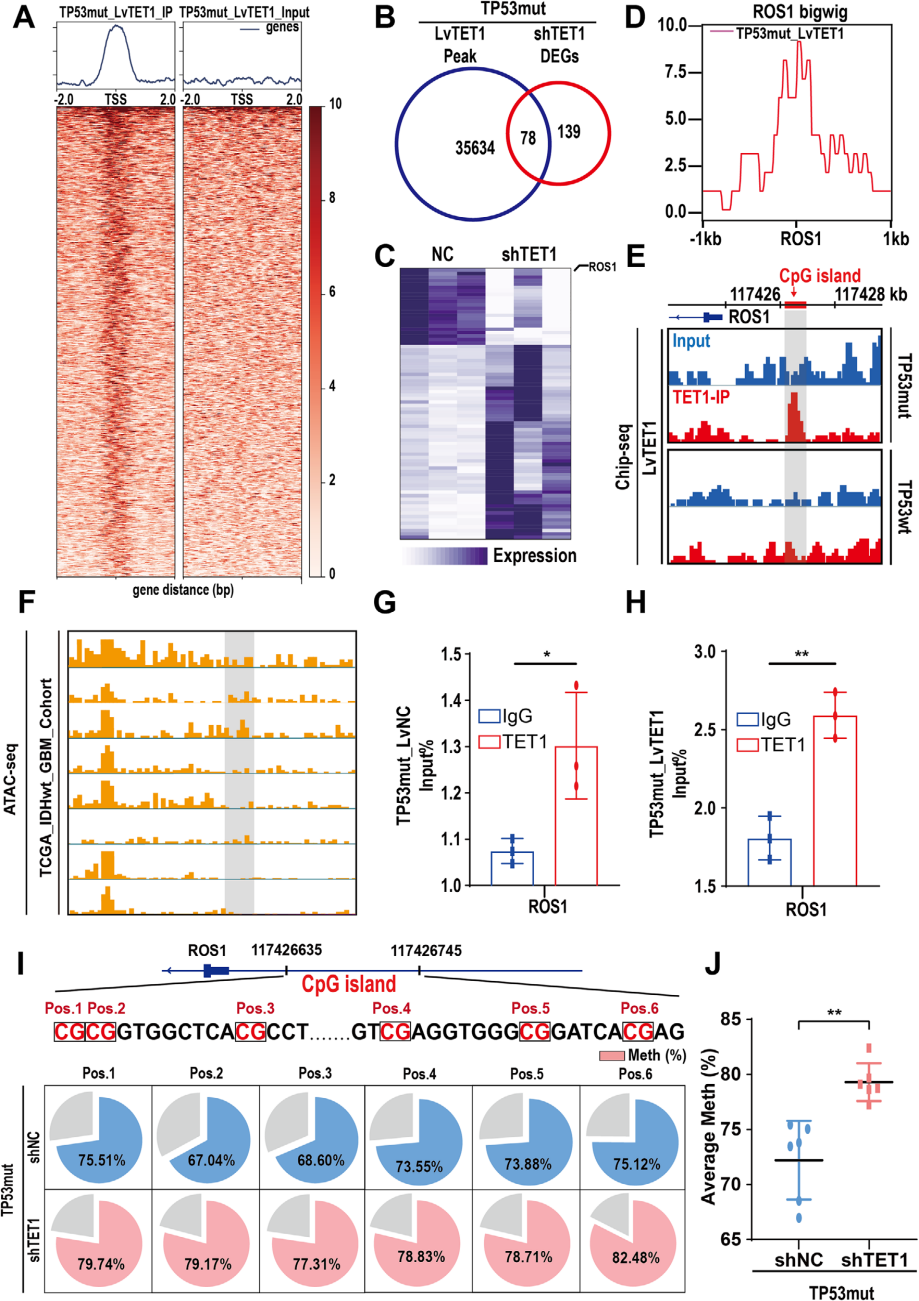
Association of TET1 with modification of the ROS1 promoter region (A) ChIP‐seq profiles of TP53mut GBM cells with TET1 overexpression showing TET1 signals at the gene promoter. (B, C) The filtering process displays the overlap of gene sets affected by TET1 by integrating ChIP‐seq and RNA‐seq. (D) Average ROS1 signal corresponding to (A). (E) Integrated Genomics Viewer (IGV) screenshot showing the results of TET1 ChIP‐seq in peaks at the genomic regions of ROS1. (F) IGV screenshot showing the results of ATAC‐seq of IDHwt GBM samples from the TCGA database in terms of peaks at the genomic regions of ROS1. (G, H) ChIP‐qPCR results showing the enrichment of TET1 at the ROS1 promoter in TP53mut GBM cells transfected with empty vector (G) or TET1‐cDNA (H). (I) Pyrophosphate sequencing profile of TP53mut GBM cells treated with shTET1 or shNC showing the methylation proportion of each CpG site in the CpG island of the ROS1 promoter region. (J) The average methylation level of the whole CpG island of the ROS1 promoter region. *n* = 3; data are presented as the means ± SDs (F, G, and J). *P < 0.05, **P < 0.01, ***P < 0.001.

To identify genes potentially affected by TET1 in TP53mut GBM, we performed RNA sequencing on TP53mut GBM cells with either TET1 knockdown (shTET1) or control (shNC). Using a significance threshold of a *p* value<0.05 and a |fold change|>1.5, we identified 217 DEGs (Figure S9D). Furthermore, GSEA revealed significant differences in enrichment scores for pathways involved in cellular senescence, the DNA damage response, and telomere organization between the shNC and shTET1 groups (Figure S9E).

By integrating the TET1‐bound target genes identified by ChIP‐seq with the genes whose expression was altered upon TET1 knockdown, we identified 78 genes whose genome exhibited significant changes in binding following TET1 knockdown in TP53mut GBM cells (*p* value<0.05 and |fold change|>1.5) (Figure 4B). Since TET1 regulated promoter demethylation and gene transcription, we specifically analyzed genes whose expression was downregulated in the shTET1 group. Among the 78 genes, ROS proto‐oncogene 1 (ROS1) was identified as the top candidate, exhibiting significant fold change in expression (|fold change| = 2) (Figure 4C). Using the MethPrimer tool, we predicted a CpG island in the ROS1 promoter. Subsequent ChIP‐seq analysis confirmed strong TET1 enrichment and binding of TET1 to the ROS1 promoter CpG island (Figure 4D,E). Furthermore, analysis of TCGA ATAC‐seq data revealed an open chromatin state at the CpG island within the ROS1 promoter, which exhibited considerable overlap with the TET1 binding regions identified by ChIP‐seq (Figure 4F). We then conducted TET1 ChIP‐qPCR at the ROS1 promoter CpG island. Our results showed significant enrichment of both endogenous (LvNC) and overexpressed TET1 (LvTET1) at this region (Figure 4G,H). These data suggest that TET1 binds to the ROS1 regulatory regions.

To determine whether TET1 regulates methylation of the CpG island in the ROS1 promoter, we conducted pyrophosphate sequencing in U251 cells treated with shNC or shTET1. The methylation percentage at individual CpG sites was greater in the shTET1 group than in the shNC group, with sites 2 and 3 being particularly affected (Figure 4I). Moreover, the average methylation percentage of all the CpG sites significantly increased upon TET1 knockdown (Figure 4J). On the basis of this evidence, we propose that TET1 binds to the CpG island in the ROS1 promoter and may be associated with CpG island methylation regulation in TP53mut GBM cells.

### 2.6 TET1 Influences ROS1 That May Contribute to ERK Phosphorylation, DNA Damage, and Cellular Senescence in TP53mut GBM Cells

We next investigated whether TET1 affects ROS1 expression. In TP53mut GBM cells, compared with the shNC control, shTET1 knockdown markedly decreased both ROS1 mRNA and protein levels (Figure 5A,B and Figure S11A). To confirm that this regulation is mediated by methylation, we treated the cells with Dec. As expected, 96‐h treatment of Dec substantially restored ROS1 expression in shTET1 cells (Figure S11B).

**FIGURE 5:**
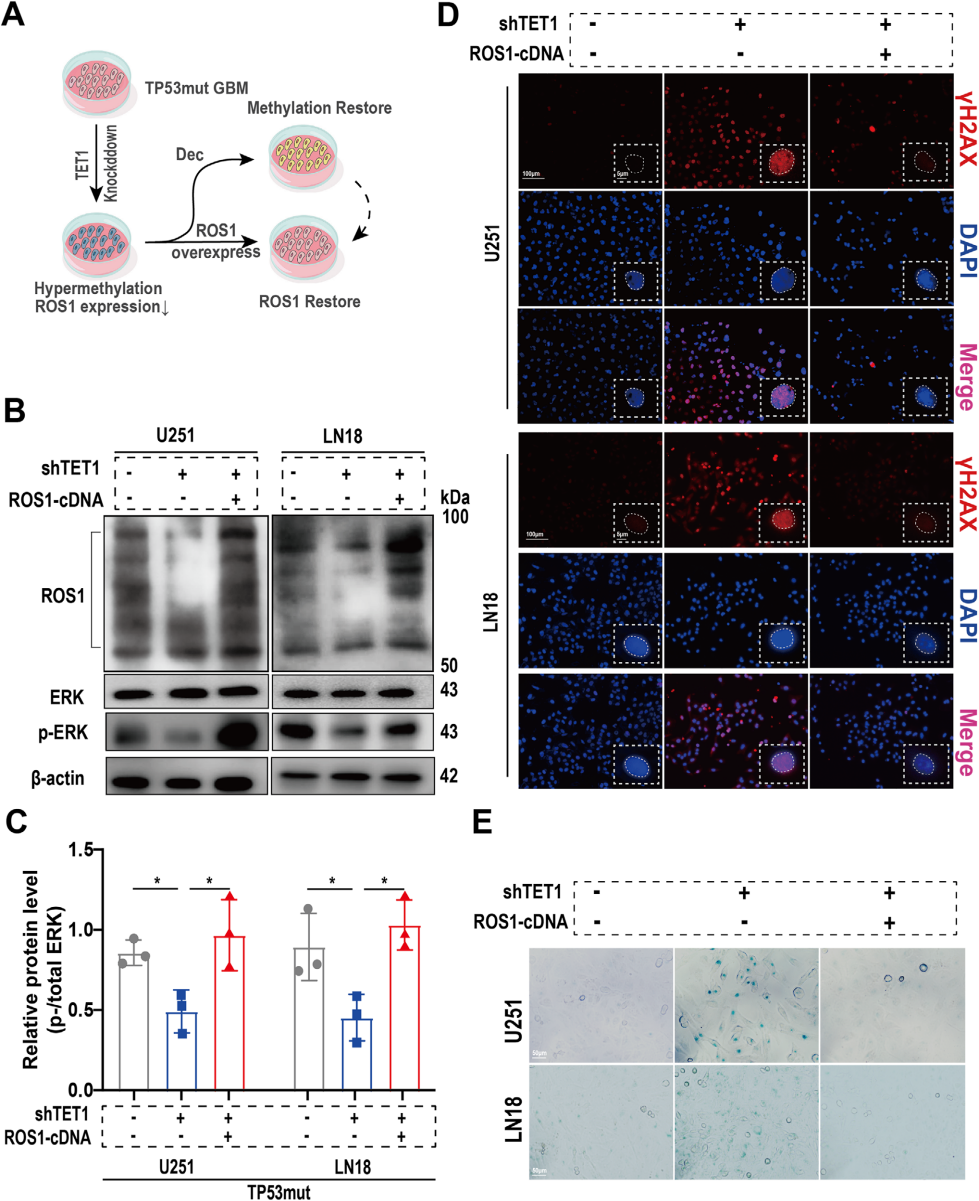
TET1 relates to ROS1 expression and ERK phosphorylation. (A) ROS1 mRNA and protein expression levels in TP53mut GBM cells with TET1 knockdown (B, C) Western blot analysis of ROS1, ERK and p‐ERK expression in U251 and LN18 cells transfected with shNC, shTET1, or shTET1+ROS1‐cDNA. The phosphorylation of ERK was quantified as the ratio of phosphorylated to total ERK. (D) Immunofluorescence staining of γ‐H2AX foci in U251 and LN18 cells transfected with shNC, shTET1, or shTET1+ROS1‐cDNA. γ‐H2AX, red; DAPI, blue. (E) SA‐β‐Gal staining of U251 and LN18 cells transfected with shNC, or shTET1+ROS1‐cDNA. *n* = 3; data are presented as the means ± SDs (C). *P < 0.05, **P < 0.01, ***P < 0.001.

ERK, a key downstream effector of ROS1, promotes therapeutic resistance upon activation, and we investigated whether TET1 regulates ERK phosphorylation through ROS1. First, we restored ROS1 expression in shTET1 TP53mut GBM cells. As expected, compared with shNC controls, TET1 knockdown significantly reduced ERK phosphorylation (pERK/ERK). This suppression was effectively reversed by ROS1 overexpression, which restored pERK/ERK levels to those transfected with an empty vector (Figure 5B,C). These results are consistent with a model in which TET1 knockdown reduces ERK phosphorylation, possibly mediated by the downregulation of ROS1.

We further assessed whether ROS1 mediates the effects of TET1 on DNA damage and cellular senescence in TP53mut GBM cells. Consistent with its role in the TET1‐ROS1 axis, compared with those in vector control‐treated cells, ROS1 overexpression significantly reduced γ‐H2AX levels and SA‐β‐Gal activity (Figure 5D,E). These results suggest that TET1 inhibition promotes DNA damage accumulation and cellular senescence while reducing ERK phosphorylation, and these effects are associated with the ROS1 pathway.

### 2.7 TET1 Knockdown Sensitizes TP53mut GBM to Cisplatin Treatment

To determine whether combining conventional chemotherapeutics with TET1 inhibition could overcome therapeutic resistance, we evaluated the viability of TP53mut GBM cells with or without TET1 knockdown subjected to four adjuvant treatment agents recommended by the NCCN Guidelines (version 2.2024) for Central Nervous System Cancers: temozolomide, procarbazine, lomustine, and cisplatin.

CCK‐8 assays conducted 48 h later revealed that compared with the shNC control, TET1 knockdown sensitized cells to the dose‐dependent growth‐inhibitory effects of cisplatin (Figure 6A,B and Figure S11C). However, TET1 knockdown did not significantly alter cellular sensitivity to temozolomide, procarbazine, or lomustine (Figure S12A–F).

**FIGURE 6:**
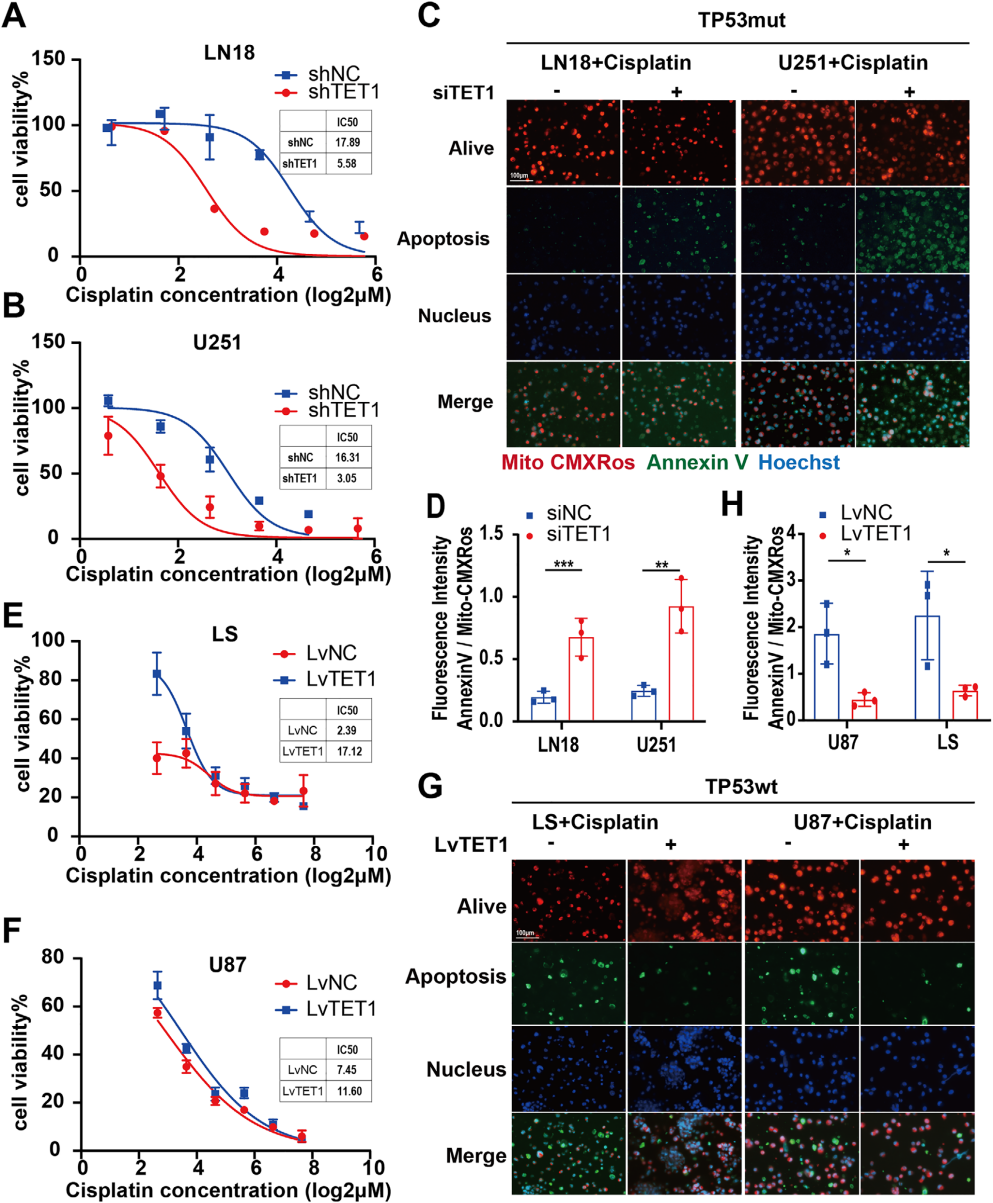
TET1 is associated with the sensitivity of GBM to cisplatin. (A, B) Dose–response curves from the CCK‐8 assay with U251 and LN18 cells transfected with shTET1 or shNC and treated with cisplatin for 48 h. (C, D) Immunofluorescence staining of U251 and LN18 cells transfected with shTET1 or shNC following exposure to 15 µM cisplatin with Annexin V (green), Mito‐CMXRos (red), and Hoechst 33342 (blue) (C) and quantification of the Annexin V/Mito‐CMXRos ratio (D). (E, F) Dose–response curves from the CCK‐8 assay with U87 and LS cells transfected with TET1 cDNA or empty vector and treated with cisplatin. (G, H) Immunofluorescence staining of U87 and LS cells expressing TET1 cDNA or the empty vector following exposure to cisplatin (7 µm for U87 cells, 2 µm for LS cells) with Annexin V (green), Mito‐CMXRos (red), and Hoechst 33342 (blue) (G) and quantification of the Annexin V/Mito‐CMXRos ratio (H). *n* = 3; data are presented as the means ± SDs (A, B, D, E, F, and H). *P < 0.05, **P < 0.01, ***P < 0.001.

To assess the impact of TET1 on cisplatin‐induced apoptosis in TP53mut GBM cells, we used a combination of Annexin V and Mito‐CMXRos staining and concurrently assessed cell viability and nuclear morphology via Hoechst staining. On the basis of these assays, 15 µm cisplatin was selected for subsequent experiments. After 48 h, compared with the siNC control, TET1 knockdown significantly increased cisplatin‐induced apoptosis, as shown by an increased Annexin V/Mito‐CMXRos ratio and greater nuclear shrinkage (Figure 6C,D). We constructed stable TET1‐overexpressing (LvTET1) TP53wt GBM lines (U87 and LS) and treated them with predetermined concentrations of cisplatin (7 and 2 µm, respectively). In contrast to TET1 knockdown, TET1 overexpression significantly attenuated cisplatin‐induced growth inhibition compared with that of the LvNC control (Figure 6E,F). After 48 h of cisplatin treatment, the apoptosis of LvTET1 cells markedly decreased, consistent with a decreased Annexin V/Mito‐CMXRos ratio and preserved nuclear morphology (Figure 6G,H).

To evaluate the role of TET1 in the response to cisplatin, we analyzed DepMap pan‐cancer cell lines stratified by the AUC (median) of cisplatin treatment. Differential expression analysis between sensitive and resistant lines revealed that the DEGs were strongly enriched in chromatin remodeling pathways (Figure S11D). Consistent with its role in regulating cisplatin sensitivity, TET1 expression was significantly elevated in resistant cell lines compared with sensitive cell lines (Figure S11E).

### 2.8 Combined TET1 Inhibitor and Cisplatin Treatment Results in Synergistic Antitumor Effects in Vitro

To examine the potential synergistic antitumor effect of TET1 inhibition and cisplatin, we treated TP53mut GBM cell lines with Bobcat339 (a specific TET inhibitor) in combination with cisplatin. Bobcat339 has been shown to alleviate hypothalamus‐associated anorexia in vivo by inhibiting TET expression in the brain [23]. After 48 h of treatment with various concentrations of both agents, cell viability was measured via a CCK‐8 assay. Analysis with SynergyFinder confirmed a synergistic effect, with ZIP scores exceeding the synergy threshold of 10 (15.74 in LN18 and 11.06 in U251; Figure 7A‐B). Following this confirmation, subsequent monotherapy and combination experiments employed 6.25 µm cisplatin and 12.5 µm Bobcat339 (Figure 7C‐D).

**FIGURE 7:**
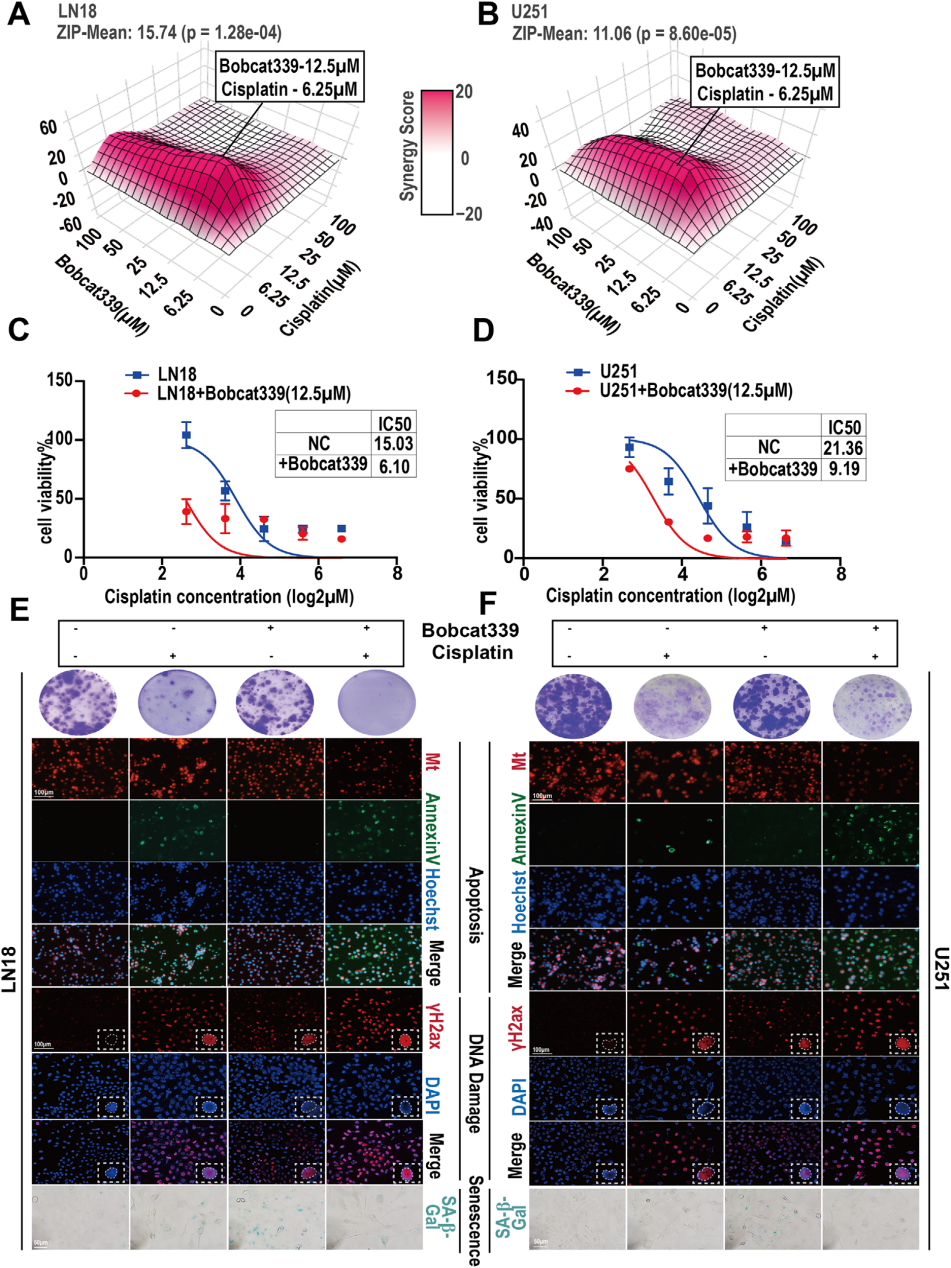
Synergistic effects of Bobcat339 and cisplatin in TP53mut GBM cell lines. (A, B) Synergy heatmaps showing ZIP scores for the Bobcat339 and cisplatin combination in U251 (A) and LN18 (B) cells. (C, D) Dose–response curves from the CCK‐8 assay with U251 and LN18 cells treated with cisplatin with or without Bobcat339 for 48 h. (E, F) Colony formation, apoptosis (Annexin V/Mito‐CMXRos), γ‐H2AX foci and SA‐β‐Gal activity in U251 (E) and LN18 (F) cells subjected to the indicated treatments. *n* = 3; data are presented as the means ± SDs (C and D).

We next assessed proliferation using colony formation assays. Although Bobcat339 monotherapy had little effect, cisplatin alone effectively suppressed colony formation. Notably, we also observed no significant difference in colony formation between shTET1‐transfected cells and shNC‐transfected cells (Figure S3F,G). The combination of both agents, however, resulted in markedly enhanced suppression compared with either treatment alone (Figure 7E,F). Similarly, in apoptosis assays, Bobcat339 alone had no effect, and cisplatin monotherapy produced only moderate apoptosis. Compared with the other treatments, the combination treatment, however, robustly increased apoptosis, as indicated by a significantly elevated Annexin V/Mito‐CMXRos ratio and more pronounced nuclear shrinkage (Figure 7E,F). These results display that Bobcat339 synergistically enhances the antiproliferative and proapoptotic effects of cisplatin in TP53mut GBM cells.

Additionally, we investigated whether Bobcat339 modulates genome fragility to sensitize TP53mut GBM cells to cisplatin. Our data revealed that both Bobcat339 and cisplatin monotherapy significantly increased the number of γ‐H2AX‐positive cells; however, compared with both the monotherapy and control groups, the combination treatment group exhibited an even greater increase in the number of TP53mut GBM cells (Figure 7E,F). Similarly, compared with the control, both Bobcat339 and cisplatin monotherapy increased the fluorescence intensity of reactive oxygen species. Compared with either monotherapy, the combination treatment, however, contributed to an even more pronounced increase in TP53mut GBM cell lines (Figure S11F). Moreover, compared with the control, both Bobcat339 and cisplatin monotherapy increased SA‐β‐Gal activity, but evaluating SA‐β‐Gal activity in the combination group was difficult because of the remarkable cell death induced by 72 h of treatment (Figure 7E,F).

### 2.9 Synergistic Antitumor Effects of the TET1 Inhibitor and Cisplatin In Vivo

To evaluate the synergistic antitumor efficacy of Bobcat339 and cisplatin in vivo, we carried out an orthotopic xenograft model using luciferase‐expressing U251 cells from NCG mice. The treatment timeline is outlined in Figure 8A. Eight days after tumor cell inoculation, the mice were randomly assigned to four treatment groups: (i) the vehicle control group, (ii) the Bobcat339 monotherapy group, (iii) the cisplatin monotherapy group, and (iv) the Bobcat339 and cisplatin combination therapy group. Tumor growth was monitored by in vivo bioluminescence imaging on days 8, 14, 20, and 24. Bobcat339 monotherapy did not significantly affect tumor growth compared with that of the control. In contrast, treatment with cisplatin alone moderately suppressed growth. Strikingly, compared with either monotherapy, the combination of Bobcat339 and cisplatin facilitated marked synergistic inhibition of tumor growth (Figure 8B–D). Furthermore, analysis of mouse weight revealed that Bobcat339 alone did not ameliorate cisplatin‐associated weight loss, whereas the combination therapy significantly alleviated this decrease compared with the monotherapy (Figure 8E).

**FIGURE 8:**
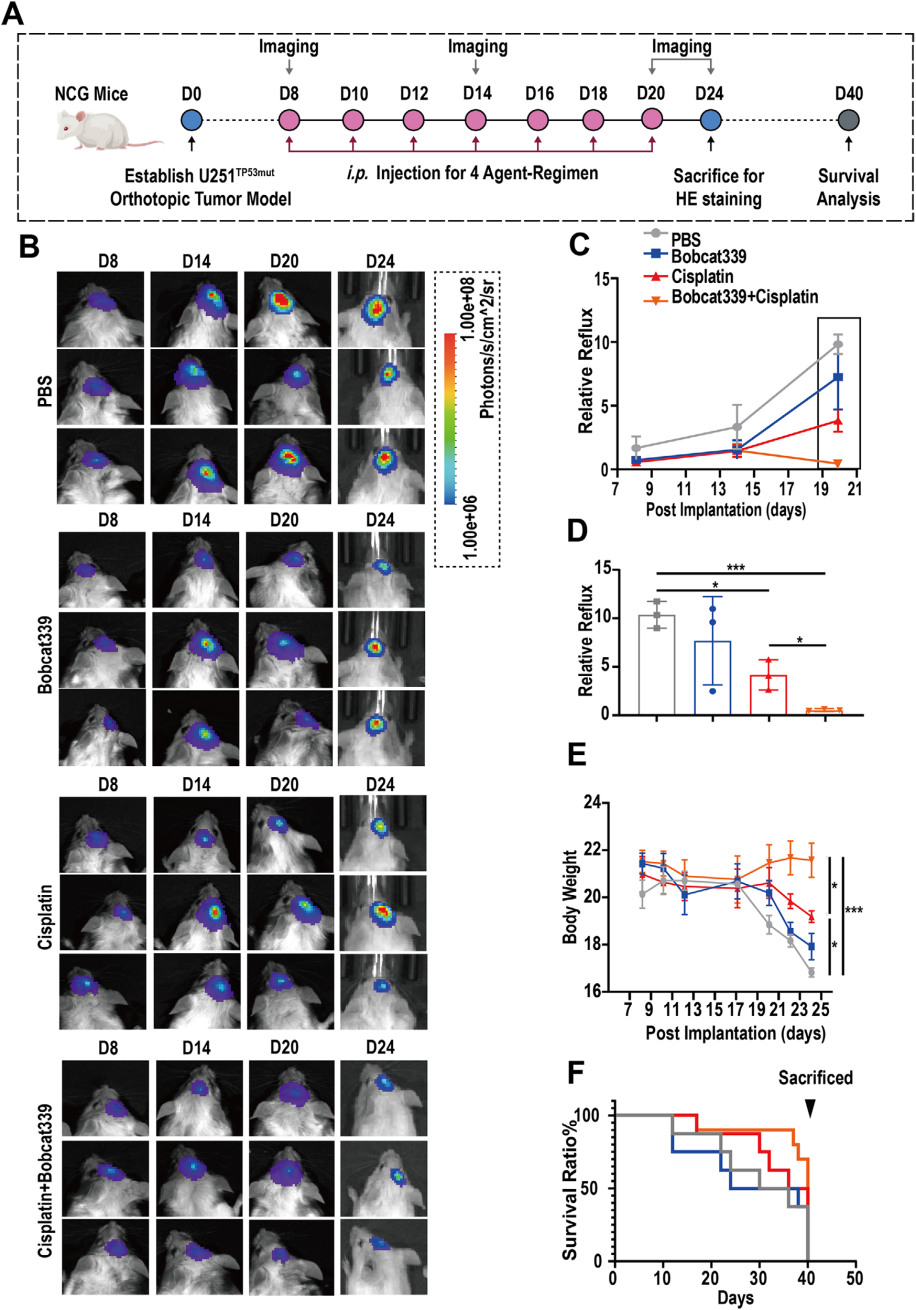
Synergistic effects of Bobcat339 and cisplatin in vivo. (A) Schematic illustration showing the timeline of the efficacy study. (B–D) Bioluminescence images of GBM tumor growth in mice treated with PBS, cisplatin, Bobcat339, or the Bobcat339+cisplatin (2 mg/kg Bobcat339, 1.5 mg/kg cisplatin), with quantification shown in (C, D) (*n* = 3). (E) Body weight changes of the mice in the indicated treatment groups until day 24 (*n* = 3). (F) Progression‐free survival analysis of mice in the indicated treatment groups until day 40 (*n* = 8). Data are presented as the means ± SDs (C–E). *P < 0.05, ***P < 0.001.

Furthermore, Kaplan–Meier analysis of progression‐free survival revealed that Bobcat339 monotherapy did not significantly extend survival time, whereas treatment with cisplatin alone had a modest survival benefit compared with the control treatment. In contrast, compared with either monotherapy, the combination of Bobcat339 and cisplatin markedly extended progression‐free survival (Figure 8F). These results collectively suggest that the Bobcat339‐cisplatin combination synergistically inhibits tumor growth, mitigates weight loss, and prolongs survival in vivo.

## 3 Discussion

TP53 is the most frequently mutated gene in cancer. In GBM, TP53mut occurs in more than 50% of cases. Despite the administration of multimodal therapies—including chemotherapy, radiotherapy, and regimens targeting the TME, such as immunotherapy, GBM patients with TP53mut invariably exhibit poor treatment responses, tumor recurrence, and poor survival outcomes [24]. Therefore, elucidating the mechanisms underlying therapeutic resistance in TP53mut GBM and identifying novel therapeutic targets are crucial for improving GBM treatment.

To identify key drivers of chemotherapy resistance in TP53mut GBM, we analyzed DEGs between TP53wt and TP53mut samples from the TCGA database. This analysis revealed that TET1 was overexpressed in TP53mut tumors. To strengthen clinical relevance, we performed IHC to quantitatively assess TET1 expression in IDHwt GBM tissues stratified by TP53 status. The results showed that TET1 expression levels were significantly higher in TP53mut GBM samples than in TP53wt samples. Given the prior links between aberrant TET1 expression and resistance to diverse anticancer agents [25, 26], we investigated its potential as a critical regulator of therapeutic resistance in this genetic context. We first examined how TP53 mutation status influences TET1 expression in GBM cell lines. Loss of wild‐type P53 transactivation capacity is a common consequence of TP53 mutation, although some TP53 mutants possess P53 GOF properties [27]. To model P53 LOF, we transfected TP53wt GBM cells with TP53‑targeting siRNA, which was associated with increased TET1 mRNA and protein expression and promoted TET1 nuclear localization. Conversely, restoring wild‑type P53 function in TP53mut cells correlated with reduced TET1 transcription, protein levels, and its nuclear localization. We note that our current data do not provide direct evidence for the mechanistic basis of how TP53 controls TET1. Additional lines of evidence (e.g., P53 occupancy at TET1 regulatory regions by ChIP‑qPCR/CUT&RUN or promoter reporter assays) are still required to establish direct transcriptional regulation. A prior study reported that transfection of wild‐type P53 into TP53mut lung cancer cells reduced TET1 promoter activity, whereas siRNA‑mediated knockdown of wild‐type P53 increased TET1 mRNA levels, demonstrating that wild‑type P53 binds the TET1 promoter to exert transcriptional repression, and that the loss of P53 relieves this repression, correlating with upregulated TET1 expression in lung cancer [18]. Taken together, our data support a model in which reduced P53 function is associated with elevated TET1 expression, but direct mechanistic evidence in GBM models remains to be established.

Genome fragility restriction as a “bulletproof vest” for cancer cells may contribute to resistance to conventional therapies [28, 29], and mutant P53 promotes this phenotype [30]. Previous studies have shown that TET1 modulates the DDR and contributes to antitumor drug resistance [31, 32]. In this study, we suggest that the cooperation of reduced P53 function and TET1 knockdown may contribute to genome fragility, consistent with the observed DNA damage accumulation, cellular senescence, telomere shortening, and increased levels of reactive oxygen species. In TP53wt GBM cells, TET1 knockdown alone was insufficient to trigger genome fragility. However, concurrent knockdown of TET1 and TP53 significantly amplified all the observed phenotypes, suggesting that these genetic perturbations may act synergistically rather than through compensatory or redundant pathways. Briefly, our findings are consistent with a model in which TP53 knockdown is associated with genome fragility (e.g., DNA damage, telomere attrition, and a predisposition to senescence), but upregulated TET1 in cancer cells could serve as a survival mechanism to counteract these processes. When TET1 is inhibited, this “survival license” seems to be revoked, and accumulated DNA damage may be converted into a lethal senescence signal, producing an anti‐tumor (genome fragility) effect probably far greater than the sum of their individual effects. The coexistence of TET1 inhibition and TP53 knockdown does not appear to involve compensatory or redundant pathway, because compensation would be expected to require another pathway to maintain function after TP53 knockdown, yet TET1 inhibition does not appear to compensate for TP53 knockdown but rather may remove a critical anti‐senescence mechanism. Likewise, redundancy would presumably require overlapping functions, whereas P53 functions in transcriptional regulation and TET1 conducts epigenetic remodeling and chromatin regulation to maintain stemness, with no substantial functional overlap based on our data.

Regarding the levels of reactive oxygen species, we found that TET1 knockdown increased reactive oxygen species levels in TP53mut GBM. However, the increase associated with TET1 knockdown could not be reversed by Dec treatment, suggesting a more complex underlying mechanism. Currently, we lack sufficient experimental data to definitively identify the reactive oxygen species source or to confirm the on‐target activity of Dec in our specific model. Previous studies have shown that TET1 affects oxidative phosphorylation and mitochondrial homeostasis, indicating that TET1 may regulate reactive oxygen species production independent of its demethylation function [33]. Thus, the complex regulatory role of TET1 in reactive oxygen species production remains to be elucidated.

Given that TET1 is an epigenetic regulator that mediates promoter CpG island demethylation, modulates chromatin accessibility, and influences gene expression, we hypothesized that its knockdown correlates with increased genome fragility through these functions [16]. Our ChIP‐seq analysis revealed that TET1 was specifically enriched at the promoter regions of signal transduction‐related genes in TP53mut GBM cells. Our integrated analysis identified ROS1 as a candidate target of TET1‐mediated epigenetic regulation. To obtain molecular evidence linking TET1 to ROS1 regulation, we performed TET1 ChIP‑qPCR of the ROS1 regulatory regions under both endogenous and exogenous conditions. We observed that TET1 was enriched at the CpG region of the ROS1 promoter, and TET1 knockdown produced corresponding changes in locus‑specific epigenetic modifications, which correlated with altered ROS1 expression. These findings are consistent with a model in which TET1 binds the CpG island in the ROS1 promoter and correlates with its hypomethylated state and transcription. Accordingly, TET1 inhibition may contribute to the ROS1 promoter hypermethylation and its transcriptional repression. Consistent with previous reports, ROS1 suppression has been shown to increase oxidative stress and DNA damage [34]. Supporting this, both ROS1 overexpression and Dec treatment appeared to attenuate these phenotypes. However, we noted that Dec‐mediated rescue of ROS1 expression alone is insufficient to establish a methylation‐dependent mechanism. Our current data could not reliably demonstrate that ROS1 regulation was strictly dependent on TET1 enzymatic function, which is a limitation of this study. Investigating the catalytic dependence of TET1 via catalytically inactive TET1 rescue experiments and the detection of 5hmc levels are important directions that we will pursue in our follow‐up investigations. Collectively, our findings suggest that TET1 binds to the ROS1 promoter region and may play a role in regulating methylation levels of this promoter, which may in turn be associated with ROS1 expression and genome fragility. Direct evidence of causation, however, requires additional experimentation.

Furthermore, we found that TET1 knockdown in TP53mut GBM cells was associated with suppressed ERK phosphorylation, and this subsequent reduction in ERK signaling was effectively reversed by rescuing ROS1 expression. In GBM, ERK activation is known to alleviate DNA damage accumulation, thereby promoting therapeutic resistance [35]. Because ROS1 has been shown to mediate ERK autophosphorylation [36], we hypothesized that TET1 knockdown impairs ERK signaling and promotes genome fragility via ROS1 suppression. Our data are consistent with this model: TET1 loss correlates with ROS1 silencing, which is accompanied by the reduction of ERK phosphorylation, and this pattern is associated with increased DNA damage and genome fragility. However, we did not perform a full set of systematic epistasis experiments (e.g., MEK/ERK inhibition or genetic manipulation, combined with ROS1 overexpression or knockdown), nor did we fully assess alternative pathways such as the AKT pathway to confirm the specific mechanism by which ROS1 regulates genome fragility. Instead, we mainly observed correlations involving ROS1 in ERK phosphorylation and genome fragility under TET1 regulation. Based on prior published studies suggesting an association between ROS1 and ERK‐dependent phenotypes, as well as the reported effects of ERK pathway on DNA damage and cellular senescence [36, 37, 38], it is possible that ROS1 could influence ERK phosphorylation and genome fragility. At present, our data support a correlation along the TET1/ROS1–ERK/DNA damage/senescence axis rather than definitive causation. Further studies are needed to rule out alternative pathways involved in ROS1 regulation of genome fragility.

Given that induced genome fragility may contribute to synergistic lethality with various anticancer modalities, we exploited the therapeutic potential of combining TET1 inhibition with adjuvant therapy agents in TP53mut GBM cells. In this study, among the adjuvant therapeutic agents, the cytotoxicity of cisplatin was uniquely increased upon TET1 inhibition, which is consistent with reports that TET1 knockdown sensitize TP53mut gastric cancer cells to cisplatin [39]. Multiple clinical trials have reported the benefit of cisplatin combination therapy for GBM patients [40, 41, 42, 43]. In accordance with the NCCN Guidelines for GBM, cisplatin‐based regimens are indicated following disease progression or intolerance to the preferred or other recommended regimens. Cisplatin‐induced apoptosis has been reported to depend on functional wild‐type P53 [44, 45]. Nonetheless, we observed that TET1 knockdown was associated with increased cisplatin‐induced apoptosis and cisplatin sensitivity in TP53mut GBM cells. Despite their status as preferred regimens for GBM treatment, the efficacy of both TMZ and lomustine has been linked to the regulation of specific DNA repair enzymes [45, 46]. Notably, in our study, the expression levels of these key repair enzymes remained virtually unaffected by TET1 knockdown. We therefore hypothesized that cisplatin would be highly susceptible to TET1 inhibition‐related genome fragility in TP53mut GBM cells. Our results indicated that the TET1 inhibitor Bobcat339 significantly increased the antitumor efficacy of cisplatin both in vitro and in vivo. Critically, this combination therapy was well tolerated, with no evidence of systemic toxicity upon analysis of the major organs (Figure S13) and on the basis that only minimal weight loss occurred during treatment. Continuous weight loss is considered a possible manifestation of GBM‐induced cachexia and has been associated with poor survival outcomes [46], and our results indicated that combination therapy alleviated GBM‐related cachexia. Together, our findings suggest that TET1 inhibition combined with cisplatin may represent a safe and effective therapy for TP53mut GBM.

Beyond direct tumor targeting, targeting the TME has emerged as a promising therapeutic strategy. As a major TME component, TAMs represent a key target in GBM. However, TP53 mutation may reduce the efficacy of TAMs‐directed therapies potentially by affecting TAMs infiltration and antigen recognition [5, 47, 48]. Notably, the TET protein family has been implicated in regulating the TAMs phenotype and the expression of immunomodulatory ligands, suggesting a role in shaping the response to immunotherapy [49, 50, 51]. Here, we observe that knocking down tumor‐intrinsic TET1 is associated with increased TAMs infiltration, differentiation (from the M0‐ to the M1‐type), M1‐type polarization, and phagocytic capacity in the context of TP53mut GBM in vitro and in vivo. Given the documented role of TET1 proteins in modulating cytokine methylation to influence TAMs function, we propose that tumor‐derived cytokines and chemokines likely serve as the mechanistic bridge linking TET1 knockdown in TP53mut GBM cells to altered TAMs biology. Our preliminary ChIP‐seq data suggest that several key factors show differential regulation upon TET1 knockdown. Together, these findings are consistent with a model in which tumor‐intrinsic TET1 may serve as a promising molecular target for reprogramming TAMs, thereby offering a potential and novel combination strategy (TET1 inhibition and TAMs targeting) for TP53mut GBM patients.

Notably, our current in vitro and in vivo experiments were designed to examine the effect of TET1 inhibition in mutant TP53 on TAMs biology, and the GOF function versus LOF effects of mutant P53 have not been dissected in this study. Furthermore, the use of immunodeficient models (nude and NSG mice) prevents us from assessing the effects TET1 inhibition in mutant TP53 on T cells, NK cells, and other myeloid subsets. Future studies using immunocompetent models (e.g., syngeneic glioma with TrP53 manipulation or a humanized immune system) will be required to fully understand the impact of TET1 knockdown on the entire TME, including the relationship between mutant P53 status and TET1‐mediated TAM regulation, and the epigenetic mechanism by which TET1 regulates TAM biology via tumor‐derived cytokines.

In summary, this study supports a potential model that TET1 may contribute to therapeutic resistance in TP53mut GBM. The direct transcriptional regulation of TET1 by TP53 in GBM requires further investigation. Furthermore, we present that TET1 inhibition correlates with genome fragility and ROS1/ERK signaling. TET1 inhibition supports a potential therapeutic vulnerability and may synergize with cisplatin, which is associated with increasing antitumor efficacy and a favorable safety profile. Additionally, we reveal a previously uncharacterized role for TET1 in modulating TAMs biology and provide new insights into targeting the TME combined with TET1 inhibition. Collectively, these findings suggest that targeting TET1 in combination with antitumor therapies may represent a promising therapeutic strategy.

## 4 Materials and Methods

### 4.1 RNA Sequencing Dataset and Analysis

The RNA sequencing dataset and the corresponding mutation information for patients with GBM were obtained from The Cancer Genome Atlas (TCGA) (the UCSC Xena browser https://gdc.xenahubs.net; TCGA Glioblastoma cohort). The raw data from the TCGA database were displayed as fragments per kilobase of exon model per million mapped fragments (FPKM). Gene set analysis on the Sangerbox platform (http://sangerbox.com/) was employed to identify the differentially expressed genes (DEGs) between TP53mut and TP53wt GBM samples, both of which lacked isocitrate dehydrogenase (IDH) mutations. We used the criteria of |fold change| > 1.5 and *p* value < 0.05 to identify the DEGs in the TCGA datasets. For gene set enrichment analysis (GSEA), genomic modifications (GO 0006304) were evaluated on the basis of Gene Ontology terms. On the basis of the gene expression profile and phenotype grouping, the minimum and maximum gene set sizes were set to 5 and 5000, respectively, and the number of resamplings was set to 1000. A *p* value < 0.05 was considered to indicate statistical significance.

### 4.2 Clinical Samples

TP53wt (*n* = 17) and TP53mut (*n* = 14) IDHwt GBM surgical specimens were obtained for the tissue microarray. All the samples were obtained from Beijing Tiantan Hospital from June 2014 to September 2019. The study was approved by the Research Ethics Committee of Beijing Tiantan Hospital (KY2014‐021‐02), and informed consent was obtained from all the patients.

### 4.3 Cell Culture

Information about TP53mut in glioma cell lines was obtained from ExPASy, a bioinformatics resource platform of the SIB Swiss Institute of Bioinformatics (https://www.expasy.org/). The human glioma cell lines U87‐MG, LN229, U373, U251, LN18 and M059K were purchased from the American Type Culture Collection and cultured in DMEM (Gibco, C11995500BT) supplemented with 10% fetal bovine serum (Gibco, 0099–141) and 1% glutamine (Gibco, 25030081) in an incubator at 37°C with 5% CO_2_. U937 cells were purchased from the American Type Culture Collection and cultured in RPMI‐1640 (Gibco, 12633020) supplemented with 10% fetal bovine serum (Gibco, 0099–141) and 1% glutamine (Gibco, 25030081) in an incubator at 37°C with 5% CO_2_. The patient‐derived GBM cell line LS was previously established and was cultured in DMEM supplemented with B27 (1:100), N2 (1:200), insulin (10 µg/mL), bFGF (10 ng/mL), EGF (10 ng/mL), and PDGF‐AB (10 ng/mL) or in DMEM with 5% fetal bovine serum, in an incubator at 37°C with 5% CO_2_. The cell lines used in this study were tested for mycoplasma contamination with a Mycoplasma Detector (Vazyme, D101‐02) and were found to be negative. All cell lines were authenticated by their respective sources. The TP53 mutation sites in different cell lines are shown in Figure S2A,B.

### 4.4 Immunohistochemical (IHC) Staining

The sections were fixed in 10% buffered formalin and embedded in paraffin. The sections cut from the paraffin‐embedded blocks were deparaffinized with xylene and ethanol. The sections were subsequently heated by microwaving in 10 mm Na citrate buffer or EDTA antigen retrieval solution for 20 min for antigen retrieval. The endogenous peroxidase of the sections was subsequently inactivated, and the sections were blocked at room temperature for one hour with goat serum. The sections were incubated overnight at 4°C with rabbit anti‐TET1 (Abcam ab191698; RRID: AB_2858250; 1:1000). A secondary antibody solution (Abcam ab6721; RRID: AB_955447; 1:5000) was added at room temperature for one hour. The final signal was developed via a DAB Horseradish Peroxidase Color Development Kit (Lablead, D2003). IHC staining was assessed by IHC Profiler.

### 4.5 Long‐Term Cell Proliferation Assays (Colony Formation Assays)

Depending on the growth rate, the cells were seeded into 6‐well plates at a density of 5×10^3^ cells per well for culture and maintained in medium containing the indicated agents for 14 days. The medium was changed twice per week. The cells were fixed with 4% formaldehyde in PBS and stained with 0.1% crystal violet diluted in water.

### 4.6 Lentiviral Infection, Small Interfering RNA (siRNA) and cDNA Transfection

Lentiviral plasmids carrying TP53‐cDNA‐Flag were purchased from Yibaike Biotechnology Co., Ltd. (Beijing) and used to overexpress TP53. Lentiviral plasmids carrying TET1 cDNA or shRNAs were used to increase or knock down the expression of TET1 and were purchased from Genechem Co., Ltd. (Shanghai) and Yibaike Biotechnology Co., Ltd. (Beijing), respectively. Small interfering RNAs (siRNAs) targeting TP53 or TET1 were used to knock down the expression of the respective genes, and ROS1‐cDNA and TET1‐cDNA were used for overexpression. Transfections were performed using Lipofectamine RNAiMAX (Invitrogen, 13778075) or Lipofectamine 3000 (Invitrogen, L3000008), according to the manufacturer's instructions. Small interfering RNAs targeting TP53 and TET1 were purchased from Tsingke Biotech Co., Ltd. (Beijing). ROS1‐cDNA was purchased from Genechem Co., Ltd. (Shanghai).

### 4.7 Western Blotting Analysis

Samples were lysed with nondenaturing lysis buffer (C1050, Applygen) supplemented with 1% protease inhibitor cocktail (Solarbio, P6730) and 1% phosphatase inhibitor (Solarbio, P1260). The samples were subjected to new flash protein AnyKD PAGE (Dakewe, 8012011, 8011071) and then transferred to polyvinylidene fluoride (PVDF) membranes (Merck Millipore Ltd. IPVH00010). The membranes were subsequently blocked with 5% nonfat milk in TBST (0.1% Tween 20) and then incubated with primary antibodies, followed by incubation with corresponding horseradish peroxidase‐conjugated secondary antibodies. The following primary antibodies were used: rabbit anti‐TET1 (Abcam ab191698; RRID: AB_2858250; 1:1000), mouse anti‐TP53 (Cell Signaling Technology 48818; RRID: AB_2713958; 1:1000), rabbit anti‐ROS1 (Cell Signaling Technology 3287; RRID: AB_2797603; 1:1000), rabbit anti‐ERK (Cell Signaling Technology 4695; RRID: AB_390779; 1:1000), and rabbit anti‐p‐ERK (Santa Cruz Biotechnology sc‐7383; RRID: AB_627545; 1:1000). Additionally, a mouse anti‐β‐actin antibody (Boao Ruijing (Beijing) Technology Development Co., Ltd., Ab1015t‐hrp; RRID: AB_3674133; 1:5000) conjugated to horseradish peroxidase was used. The following secondary antibodies were used: anti‐rabbit IgG with HRP (Abcam ab6721; RRID: AB_955447; 1:5000) and anti‐mouse IgG with HRP (Abcam ab205719; RRID: AB_2755049; 1:5000). Specific protein bands were visualized via enhanced chemiluminescence reagents (NCM Biotech, P10300) on an Amersham Imager 600 (GE). The final data were subjected to grayscale scanning and semiquantitative analysis via ImageJ software (https://imagej.nih.gov/ij/download.html).

### 4.8 Immunofluorescence (IF) Staining

GBM cells were seeded onto round coverslips in 24‐well plates and treated with the indicated agents. The cells were then fixed with 4% paraformaldehyde for 30 min and rinsed three times with PBS. The cells were treated with 5% glycine for 5 min to reduce autofluorescence and permeabilized with 0.3% Triton X‐100 for 15 min. The coverslips were then incubated with 1% bovine serum albumin in 0.3% Triton X‐100 for one hour and then washed three times with PBS. Next, the coverslips were incubated overnight with the following primary antibodies: rabbit‐derived γ‐H2AX (Abcam ab81299, RRID: AB_1640564, 1:150), rabbit‐derived TET1 (Abcam ab191698, RRID: AB_2858250, 1:150), rat‐derived CD86 (Abcam ab119857, RRID: AB_10902800), rabbit‐derived CD206 (Abcam ab300621, RRID: AB_2935881) and rabbit‐derived CD68 (Abcam ab213363, RRID: AB_2801637, 1:200) antibodies. After washing, the cells were incubated with Cy3‐conjugated anti‐rabbit IgG (Beyotime P0183, RRID: AB_3674131) and FITC‐conjugated anti‐rabbit IgG (Beyotime P0186, RRID: AB_3674132), AF594‐conjugated anti‐rabbit IgG (Beyotime, A1200, RRID: AB_3741722), FITC‐conjugated anti‐mouse IgG (Beyotime, A0568, RRID: AB_2893016), Cy3‐conjugated anti‐mouse IgG (Beyotime, P0183, RRID: AB_2923334), and FITC‐conjugated anti‐rat IgG (Beyotime, A1113, RRID: AB_3750272) at room temperature for one hour, followed by three washes with PBS. The coverslips were stained with DAPI for 5 min and then washed three times with PBS. Afterward, the coverslips were transferred to carrier glass with antifade mounting medium. Images were acquired using an Axio Observer Z1 microscope and subjected to quantification.

### 4.9 Detection of Senescence‐Associated Beta‐Galactosidase (SA‐β‐gal)

SA‐β‐gal activity, a widely used marker of cellular senescence, was measured with a senescence‐associated beta‐galactosidase assay kit (Beyotime, C0602) following the manufacturer's instructions. Briefly, cells were seeded in 6‐well plates and cultured for 72 h. Afterward, the cells were fixed for 15 min with the fixative solution provided in the kit and rinsed three times with PBS. The cells were incubated with staining solution overnight in a water bath at 37°C. The cells that appeared blue under the microscope were identified as positively stained cells.

### 4.10 Telomere Q‐FISH

Cells transfected with shNC or shTET1 were passaged to approximately the 10th generation for telomere detection. The cells were cultured with the indicated agents and 0.2 mg/mL colcemid for two hours to enrich the population of cells at metaphase. Harvested cells were treated with a 75 mm KCl solution, fixed with Carnoy's fixative solution (methanol: acetic acid, 3:1), and spread onto clean glass slides. The slides were then washed with PBS and fixed with 4% paraformaldehyde, followed by incubation with 1 mg/mL pepsin (pH 2.0). The slides were then postfixed with 4% paraformaldehyde, dehydrated in an ethanol series, and air dried. Telomeres were denatured for 3 min at 80°C and hybridized with a telomere‐specific peptide nucleic acid (PNA) probe for 4 h at room temperature. Chromosomes were counterstained with DAPI. Telomere images were captured by a confocal microscope with fixed exposure parameters. For quantitative measurement of telomere length, fluorescence intensity was analyzed via the Telometer package in ImageJ.

### 4.11 Telomere Length qPCR Quantification

Telomere length quantification was carried out as previously described [52]. Total DNA was isolated via a SteadyPure Universal Genomic DNA Extraction Kit (Accurate Biology, AG21009) and diluted to 1.5 ng/µL. Relative telomere length was determined using the 2^−ΔΔCT^ method with IFNB1 as the reference gene.

### 4.12 Quantitative Real‐Time PCR Assay

Total RNA was isolated via a SteadyPure Quick RNA Extraction Kit (Accurate Biology, AG21023) and reverse transcribed to generate cDNA with an S6 Super qPCR RT Kit (Science Tool, S6166). The cDNA was amplified via Power SYBR Green (Applied Biosystems, 4367659) with Quant Studio 5 (Applied Biosystems). The amplification program was as follows: initial denaturation at 95°C for 30 s, followed by 40 cycles at 95°C for 15 s and 60°C for 60 s. The average fold change in expression was calculated on the basis of the threshold cycle for ACTB mRNA. Relative gene expression was determined via the 2^−ΔΔCT^ method. The primer sequences used for the qPCR are listed in the Table S1.

### 4.13 Reactive Oxygen Species Detection

The reactive oxygen species level was measured out with a Reactive Oxygen Species Assay Kit C1300 (Beijing Solarbio Science and Technology, Beijing, CN). Briefly, cells were harvested and resuspended. After centrifugation, the cell pellet was collected, and dihydroethidium probe reagent was added followed by incubation for 30 min. The cells were then centrifuged again, and the pellet was resuspended. Fluorescence micrographs were obtained using an inverted fluorescence microscope. The fluorescence intensity, reflecting the level of reactive oxygen species, was quantified.

### 4.14 Detection of the TAMs Phagocytic Capacity

Lentiviral plasmids carrying GFP were used to quantify GBM tumor cells treated with shNC or shTET1 and were purchased from Genechem Co., Ltd. (Shanghai). To obtain macrophages, U937 cells were cocultured with 100 ng/mL phorbol 12‐myristate 13‐acetate (PMA) for 48 h. GBM tumor cells expressing GFP were cocultured with macrophages for 48 h to determine the phagocytic capacity of the TAMs.

### 4.15 Flow Cytometry Analysis

The cells were harvested with 0.25% trypsin, washed with cell staining buffer (BioLegend, 420201), and incubated with Human TruStain FcX (BioLegend, 422301) at room temperature. The cells were subsequently incubated with anti‐CD68 (BioLegend, 137008), anti‐CD86 (BioLegend, 381009), anti‐CD206 (BioLegend, 321105) or isotype control antibodies (BioLegend, 400322) at 4°C for 15 min. The signals were detected on a flow cytometer (BD Accuri C6 Plus), and data were analyzed with FlowJo software. The cells were gated on the basis of forward scatter (FSC) and side scatter (SSC), and then the CD68 and GFP fluorescence were analyzed.

### 4.16 Transwell Infiltration Assay

The infiltration ability of TAMs was determined using 24‐well Transwell chambers precoated with Matrigel. Approximately 2×10^4^ macrophages differentiated from U937 cells in complete RPMI‐1640 medium were placed into the upper chamber. The conditioned medium obtained from GBM cells (cultured for 48 h) was added to the lower chamber. After 24 h at 37°C, noninfiltrating cells on the upper surface were gently removed with a cotton swab. Cells that had infiltrated through the Matrigel‐coated membrane were fixed in 4% paraformaldehyde, stained with 0.1% crystal violet (Beyotime, C0121), and photographed under a phase‐contrast microscope.

### 4.17 RNA‐Seq

Total RNA was extracted from the samples by TRIzol reagent (Invitrogen). The RNA quality was assessed with an Agilent 2200 instrument, and samples were stored at −80°C. RNA with an RNA integrity number (RIN) > 7.0 was deemed acceptable for cDNA library construction. cDNA libraries were constructed for each RNA sample via the VAHTS Universal V6 RNA‐seq Library Prep Kit for Illumina (Vazyme, Inc.) according to the manufacturer's instructions. In general, the protocol consists of the following steps: Poly‐A‐containing mRNA was purified from 1 µg of total RNA via oligo(dT) magnetic beads and fragmented into 200–600 bp fragments via treatment with divalent cations at 85°C for 6 min. The cleaved RNA fragments were used for first‐ and second‐strand complementary DNA (cDNA) synthesis. A dUTP mixture was used for second‐strand cDNA synthesis, which allowed for the removal of the second strand. The cDNA fragments were end repaired, A‐tailed, and ligated with indexed adapters. The ligated cDNA products were purified and treated with uracil DNA glycosylase to remove the second‐strand cDNA. The purified first‐strand cDNA was enriched via PCR to create cDNA libraries. The libraries were subjected to a quality control assessment with an Agilent 2200 instrument and sequenced via DNBSEQ‐T7 on a 150 bp paired‐end run.

We applied the DESeq2 algorithm to identify the differentially expressed genes on the basis of the count data that were filtered as having a count >100. After the significance analysis, DEGs were selected based on the criteria of a |fold change|>1.5 and a *p* value<0.05. GSEA was performed as described above.

### 4.18 ChIP‐Seq and ChIP‐qPCR

To identify as many TET1‐influenced genes as possible, we generated TET1‐overexpressing cell lines by transfecting TP53mut and TP53wt GBM cells with TET1 cDNA. TET1‐overexpressing U251 and LN18 cells were mixed into one group, named TP53mut GBM, and U87 and LS cells were mixed into one group, named TP53wt GBM.

Cells were crosslinked with 1% formaldehyde (final concentration) for 10 min by inverting the flasks at room temperature and quenched with 0.125 m glycine for 5 min. The cell pellets were washed repeatedly in PBS and then stored at −80°C. The pellets were lysed in lysis buffer (50 mm HEPES, 150 mm NaCl, 1 mM EDTA, 0.1% SDS, 0.1% sodium deoxycholate, 1% Triton X‐100, and supplemented with protease inhibitor cocktail) for 10 min. After centrifugation, the supernatant was discarded, and the pellet was lysed in lysis buffer and subjected to sonication. Sheared chromatin was incubated with the primary antibody (anti‐TET1, Thermo, GT1462, RRID: AB_11172316) bound to Pierce Protein A/G Agarose Beads (Thermo Fisher Scientific, Inc.) overnight, followed by elution and reverse cross‐linking at 65°C overnight. TE buffer (10 mm EDTA) was added to the DNA elution buffer, followed by RNase treatment (0.5 mg/mL) at 37°C for 30 min and proteinase K treatment (0.3 mg/mL) at 51°C for one hour, after which the DNA was isolated and subsequently purified. Immunoprecipitated DNA was used to construct Illumina sequencing libraries following the manufacturer's instructions and sequenced on an Illumina NovaSeq 6000 sequencing platform or used for qPCR as described above.

After the raw data in FastQC were evaluated, low‐quality reads were removed by using Trimmomatic, and the cleaned ChIP‐seq reads were mapped to the reference genome using BWA software. Read counts were normalized to reads per million mapped reads (RPM). Peak calling was performed using MACS2 with a *p* value<0.05. The regions of the peaks were defined on the basis of the annotations of the reference genome. Bigwig files were generated from BAM files using bamCoverage function in deepTools. Track diagrams were generated using Integrated Genomics Viewer (IGV) for visualization. The peak‐by‐sample count matrix was obtained by counting the number of reads overlapping with each peak.

### 4.19 ATAC‐Seq Analysis

The BigWig files of ATAC‐seq data were acquired from the TCGA (https://gdc.cancer.gov/about‐data/publications/ATACseq‐AWG; GDC GBM cohort). After the data were filtered, we obtained ATAC‐seq data for eight IDHwt GBM samples. The BigWig files were uploaded to IGV for visualization.

### 4.20 Pyrophosphate Sequencing

Total DNA was extracted from the samples using a Rapid Animal Genomic DNA Isolation Kit (Sango, B518221). DNA quality was assessed with an Agilent 2200 instrument, and the samples were stored at −80°C. Bisulfite modification was performed using the Column DNA Methylation Bisulfite Conversion Kit (Sango, B618603) according to the manufacturer's recommendations. After the target sequence was amplified using a PCR instrument, the product was subjected to pyrophosphate sequencing with PyroMark Q48 Autoprep. Finally, this method yielded a quantitative value for the percentage of methylated alleles for each of the investigated CpG sites. To define cases with methylated vs. unmethylated ROS1 promoters, the mean value of the methylation percentage obtained at each of the investigated CpG dinucleotides was calculated.

### 4.21 Cell Viability Assay

Cell viability was assessed via a Cell Counting Kit‐8 (CCK‐8) assay (Biosharp, BS350A). Approximately 4×10^3^ cells were seeded in each well of 96‐well plates. The cells were allowed to attach overnight. The cells were subsequently treated with various agents. Cell viability was evaluated with a CCK‐8 assay. The absorbance values were determined at 450 nm on a Synergy H1 MFD spectrophotometer (BioTek).

### 4.22 Apoptosis Detection

Apoptosis was detected with a Mitochondrial Membrane Potential Detection Kit (C1071; Beyotime, Shanghai, China). The cells were resuspended in PBS and fixed for 15 min with the specific agents provided in the kit. A total of 5 × 10^4^ cells were collected by centrifugation and resuspended in 188 µL of mixture containing of the following reagents: 2 µL of MitoTracker Red CMXRos, 5 µL of Annexin V‐FITC, and 5 µL of Hoechst 33342. After mixing, the cells were incubated for 30 min at 37°C. The resuspended cells were imaged with an Axio Observer Z1 microscope and quantified.

### 4.23 Synergy Determination with SynergyFinder

The cells were seeded into 96‐well plates at 4×10^3^ cells per well and further treated as described below. Either single agents (Bobcat339 or cisplatin) or combinations of agents were used at the concentrations indicated for the cytotoxicity assay above. The concentration gradient of Bobcat339 or cisplatin was predetermined by the IC50 value of each agent, and cell viability was analyzed at a constant dilution ratio of the two inhibitors. After 48 h of treatment, cell viability was evaluated with a CCK‐8 assay as described above. The online SynergyFinder+ software (https://synergyfinder.org/) was used to calculate drug synergy scores with the inhibition index via response surface modeling and zero interaction potency (ZIP) calculation methods. ZIP synergy scores greater than 0 were considered synergistic (red regions), and scores greater than 10 were considered strongly synergistic [53]. 3D heatmaps of drug combination responses were generated to assess the therapeutic significance of the combination.

### 4.24 Subcutaneous Xenograft Mouse Model Experiments

BALB/c nude mice were anesthetized, completely randomly assigned to two groups, and received a subcutaneous injection of 1×10^7^ U251 cells treated with shNC or shTET1 in 100 µL of PBS into the right dorsal flank. Pre‑established experimental end point criteria were as follows: (1) mice with tumors exceeding 10% of their body weight, (2) mice showing signs of cachexia (>20% weight loss), and (3) death. No animals at the study endpoint were excluded from the final analysis. The mice were humanely euthanized when the tumors were visible after eight days at the predefined experimental endpoint. The tumor samples were surgically excised and photographed for subsequent analyses.

### 4.25 Orthotopic Xenograft Mouse Model Experiments

NCG mice were anesthetized with isoflurane and placed in a stereotactic head frame, and a burr hole was drilled on the coronal suture 2.0 mm lateral (right) to the bregma. U251 cells (2×10^5^ cells in 5 µL of PBS) expressing luciferase via lentiviral infection (U251‐luc) were slowly injected with a Hamilton syringe into the brain at a depth of 3.5 mm. Bone wax was used to close the hole, and the wound was sutured. Eight days after surgery, the NCG mice were imaged via an IVIS system 10 min after 150 mg/kg d‐luciferin was intraperitoneally injected. The bioluminescence intensity was quantified with Living Image software provided by the same manufacturer. The weights of the mice were measured at regular intervals. Three mice in each group were completely randomly selected to be sacrificed on day 24 after surgery for H&E staining to evaluate systemic toxicity. The remaining mice were sacrificed on day 40 after surgery, and progression‐free survival was determined.

### 4.26 Administration of Agents In Vivo

Bobcat339 was suspended in 1% DMSO in PBS, and cisplatin was suspended in PBS. When tumor growth was measurable (eight days after injection), the NCG mice were randomly assigned to four groups according to the completely randomized design: vehicle (1% DMSO in PBS), Bobcat339 (2 mg/kg in a final formulation of 1% DMSO in PBS), cisplatin (1.5 mg/kg in a final formulation of PBS), or both in combination (2 mg/kg Bobcat339 and 1.5 mg/kg cisplatin in a final formulation of 1% DMSO in PBS) intraperitoneal injections were administered over a total of 14 days (every two days for a total of 7 injections). Pre‑established experimental end point criteria were as follows: (1) mice with tumors exceeding 10% of their body weight and (2) died from causes unrelated to the treatment. No animals at the study endpoint were excluded from the final analysis based on these criteria. At the experimental end point, the mice were anesthetized with isoflurane and subsequently sacrificed by cervical dislocation prior to tissue collection.

### 4.27 Statistics

All statistical values were calculated with GraphPad Prism 9.0 (GraphPad Software, Inc.). One‐way ANOVA was used for comparisons between more than two groups. Two‐tailed unpaired Student's *t* tests were performed to analyze the differences between two groups. All the quantitative data are presented as the mean ± standard deviation (SD) from at least three biological replicate samples or independent experiments per data point. A *p* value less than 0.05 was considered to indicate statistical significance, and multiple comparisons were not applied due to the limited sample size. Sample or experiment size (n) is indicated in each figure legend. The investigators responsible for assessing the in vivo experimental outcomes (e.g., tumor measurements, weight measurements, histological scoring, IF staining, etc.) were blinded to group allocation.

## Author Contributions

**Zhuonan Pu**: conceptualization, methodology, writing – review and editing, investigation, supervision, project administration, writing – original draft, formal analysis. **Jinqiu Liu**: methodology, investigation, project administration. **Yuxuan Deng**: writing – review and editing, methodology, visualization. **Shuyu Hao**: funding acquisition, methodology, resources. **Xiaoli Zhang**: validation. **Mingxu Yang**: project administration. **Chao Guo**: investigation. **Chao Du**: project administration, software. **Yingdan Chen**: data curation. **Tai Sun**: resources. **Nan Ji**: resources, supervision. **Zhengping Zhuang**: writing – review and editing, supervision. **Jie Feng**: supervision, writing – review and editing, writing – original draft, conceptualization, funding acquisition, project administration, data curation, resources.

## Funding

This work was supported by the Beijing Municipal Health Commission (No. 11000026T0000037102440‐2 to J. F), the Beijing Natural Science Foundation (No. 7262012 to J. F), the National Natural Science Foundation of China (No. 81872052 to S. H), and the Beijing Nova Program (No. 20240484672 to S. H).

## Ethics Statement

The study was approved by the Research Ethics Committee of Beijing Tiantan Hospital (no. KY2014‐021‐02). All the animal protocols were approved by the Animal Welfare Ethics Committee of Beijing Neurosurgical Institute (no. BNI202503008).

## Consent

Informed consent was obtained from all enrolled subjects. The study was carried out in full compliance with all principles of the World Medical Association Declaration of Helsinki.

## Clinical Trial Registration

Not applicable

## Conflicts of Interest

The authors declare that no conflicts of interest exist.

## Supporting information




**Supporting File 1**: advs76517‐sup‐0001‐SuppMat.docx.

**Supporting File 2**: advs76517‐sup‐0002‐TableS1.xlsx.

## Data Availability

The datasets used and/or analyzed during the current study are available in the supplementary data or can be obtained from the corresponding author upon reasonable request.
