# Supplementary material for: TET1 Inhibition Promotes Therapeutic Sensitivity in TP53‐Mutant GBM by Influencing Genome Fragility and Altering TAMs Biology
Source: Adv Sci (Weinh). 2026 Jul 11:e76517. Online ahead of print. doi: 10.1002/advs.76517 (PMC13355933; doi:10.1002/advs.76517)
Supplement: Supplementary file 1 — Supporting File 1: advs76517‐sup‐0001‐SuppMat.docx. [file ADVS-9999-e76517-s002.docx]

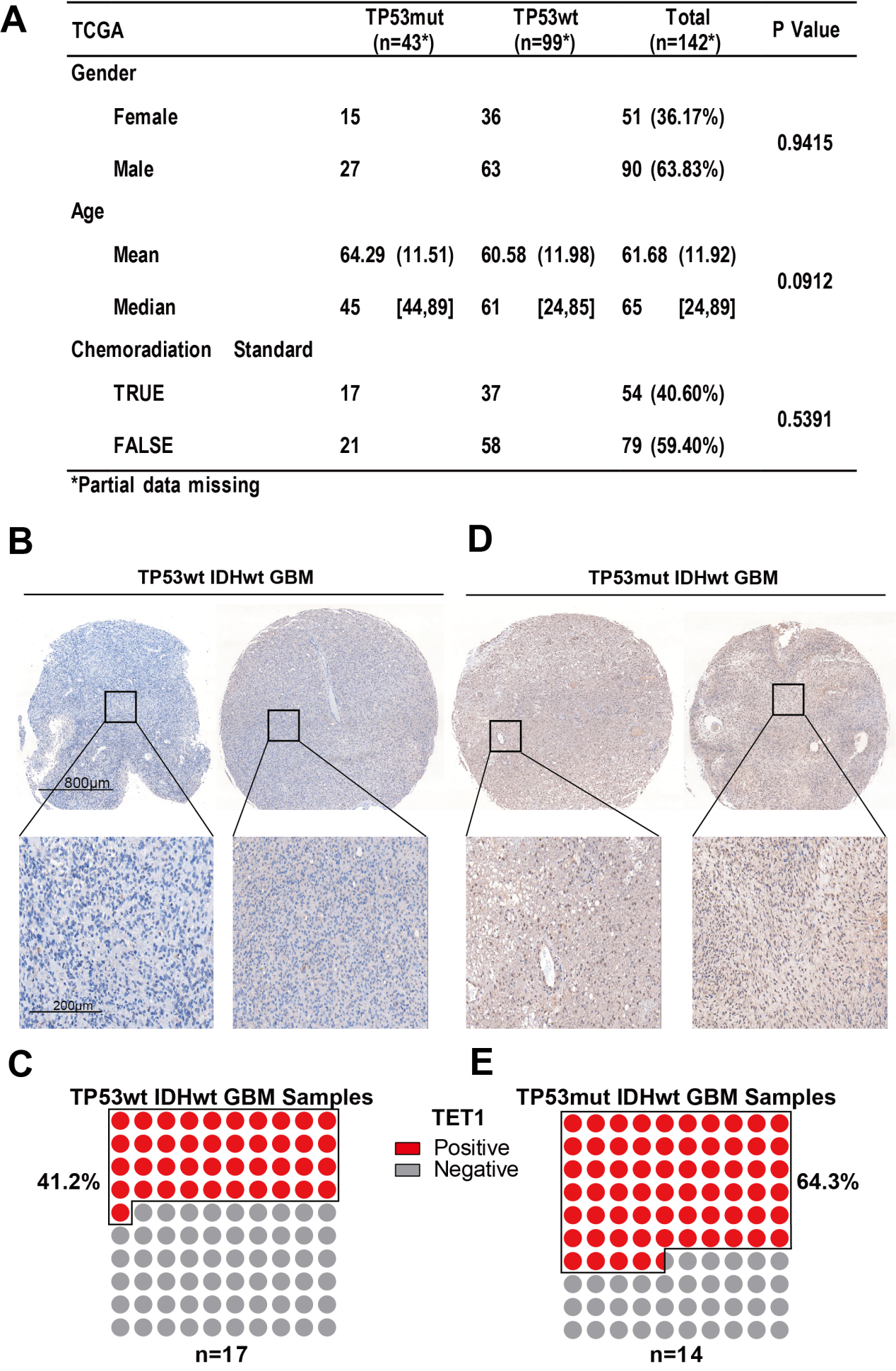


**Figure S1.** (A) Clinical features of TP53mut and TP53wt GBM samples from the TCGA dataset. (B-E) IHC staining images of TET1 in TP53wt IDHwt GBM (n=17) (B) and TP53mut IDHwt GBM (n=14) samples (D) and quantification of the proportion of TET1-positive samples in (C) and (E), respectively.


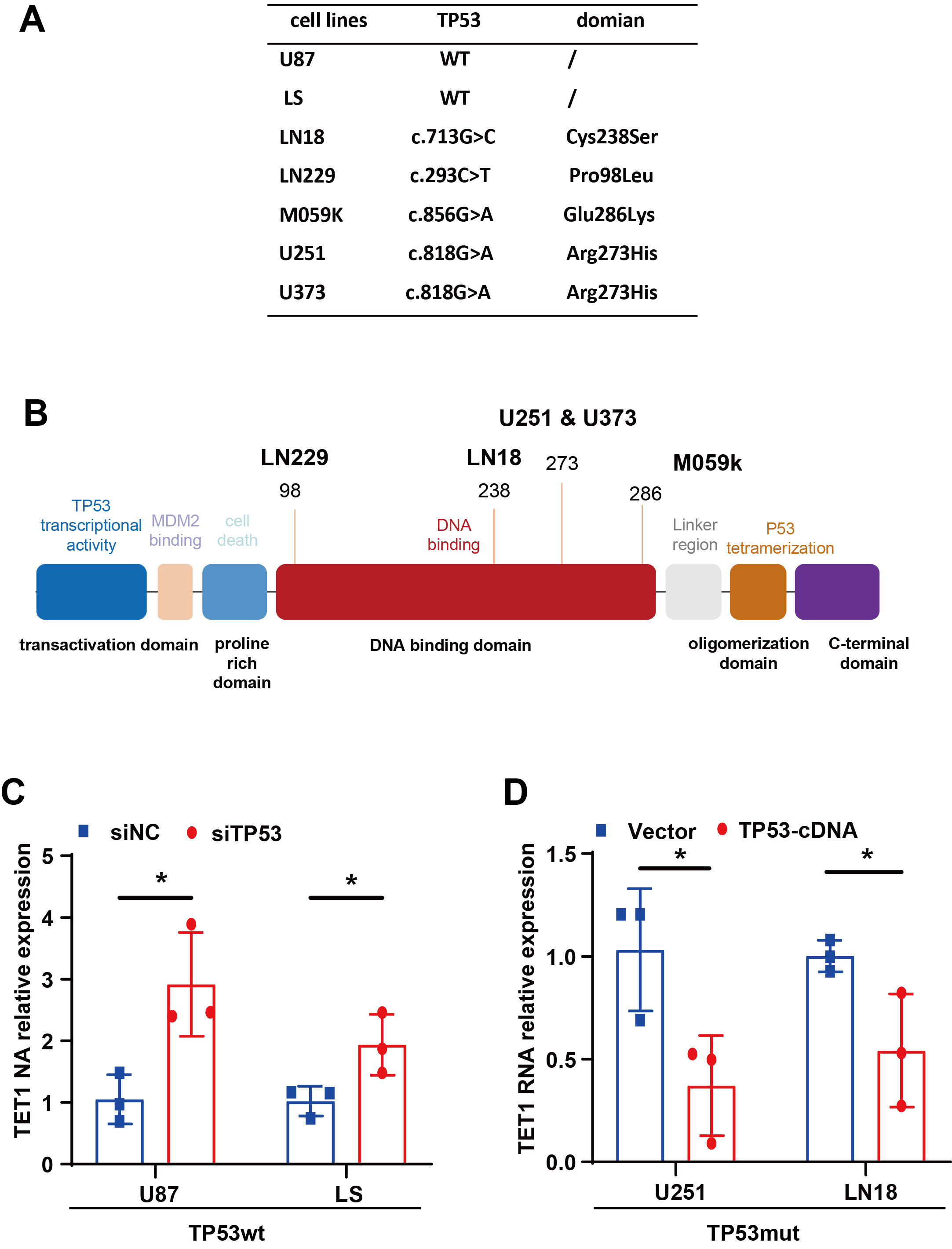


**Figure S2** (A) TP53 mutation site and corresponding changes in amino acid residues. (B) Linear schematic representation of the main domains in the P53 protein. (C) qPCR analysis and quantification of TET1 mRNA levels in TP53wt U87 and LS GBM cells transfected with siTP53 or siNC. (D) qPCR analysis and quantification of TET1 mRNA levels in TP53mut U251 and LN18 GBM cells transfected with TP53-cDNA or empty vector. n=3; data are presented as the means ± SDs (C, and D). *P<0.05.


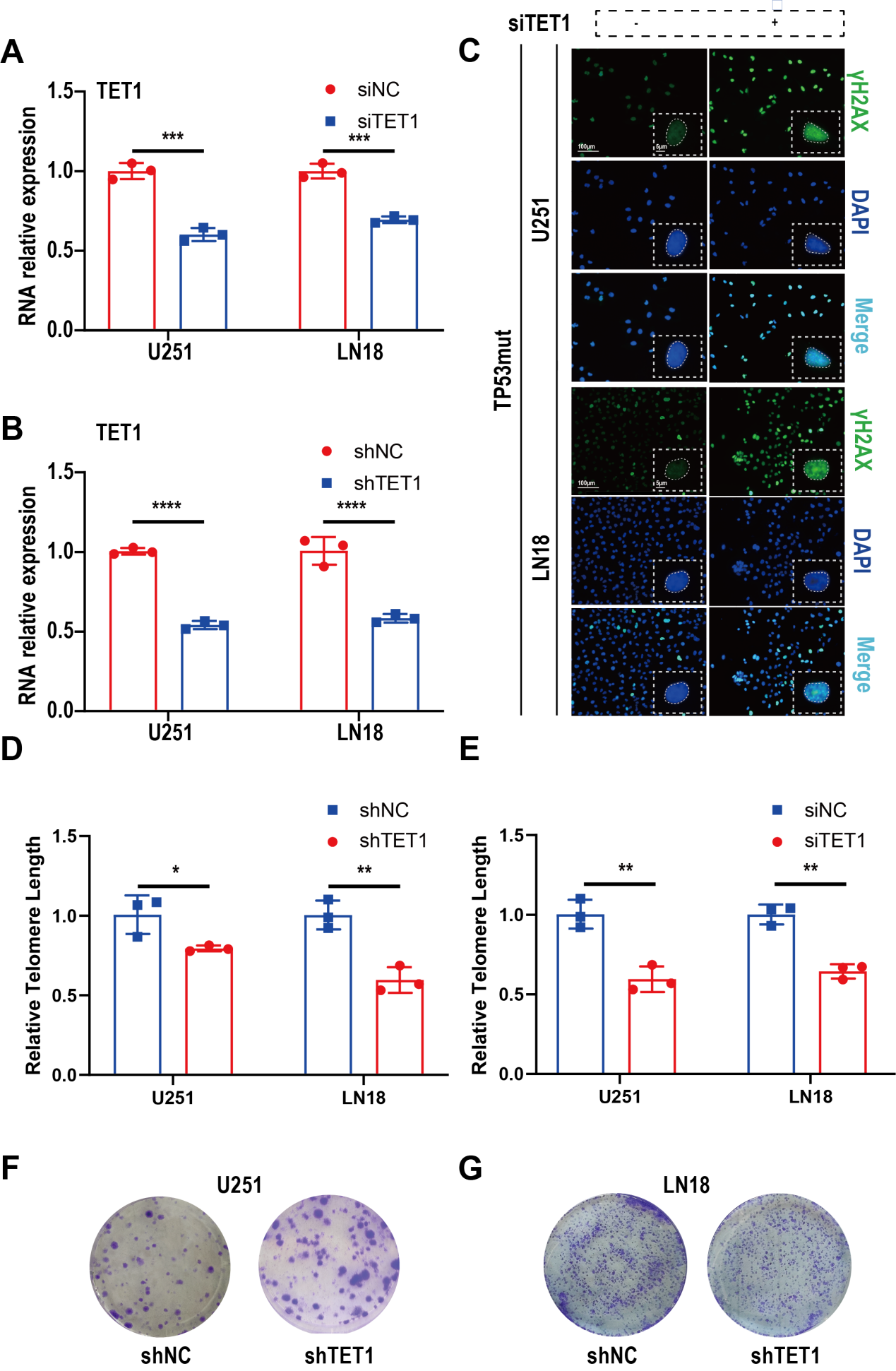


**Figure S3 TET1 knockdown relates to DNA damage and telomere shortening.** (A) qPCR analysis and quantification of TET1 mRNA levels in U251 and LN18 cells transfected with siTET1 or siNC. (B) qPCR analysis and quantification of TET1 mRNA levels in U251 and LN18 cells transfected with shTET1 or shNC (n=3). (C) Immunofluorescence staining images and quantification of γ-H2AX foci in U251 and LN18 cells transfected with siTET1 or siNC (n=4). γ-H2AX, green; DAPI, blue. (D) qPCR analysis and quantification of telomere length in U251 and LN18 cells expressing shTET1 or shNC (n=3). (E) qPCR analysis and quantification of telomere length in U251 and LN18 cells transfected with siTET1 or siNC (n=3). (F–G) Results of the colony formation assay with U251 (F) and LN18 (G) cells transfected with shTET1 or shNC. Data are presented as the means ± SDs. *P<0.05, **P<0.01, ***P<0.001, ****P<0.0001.


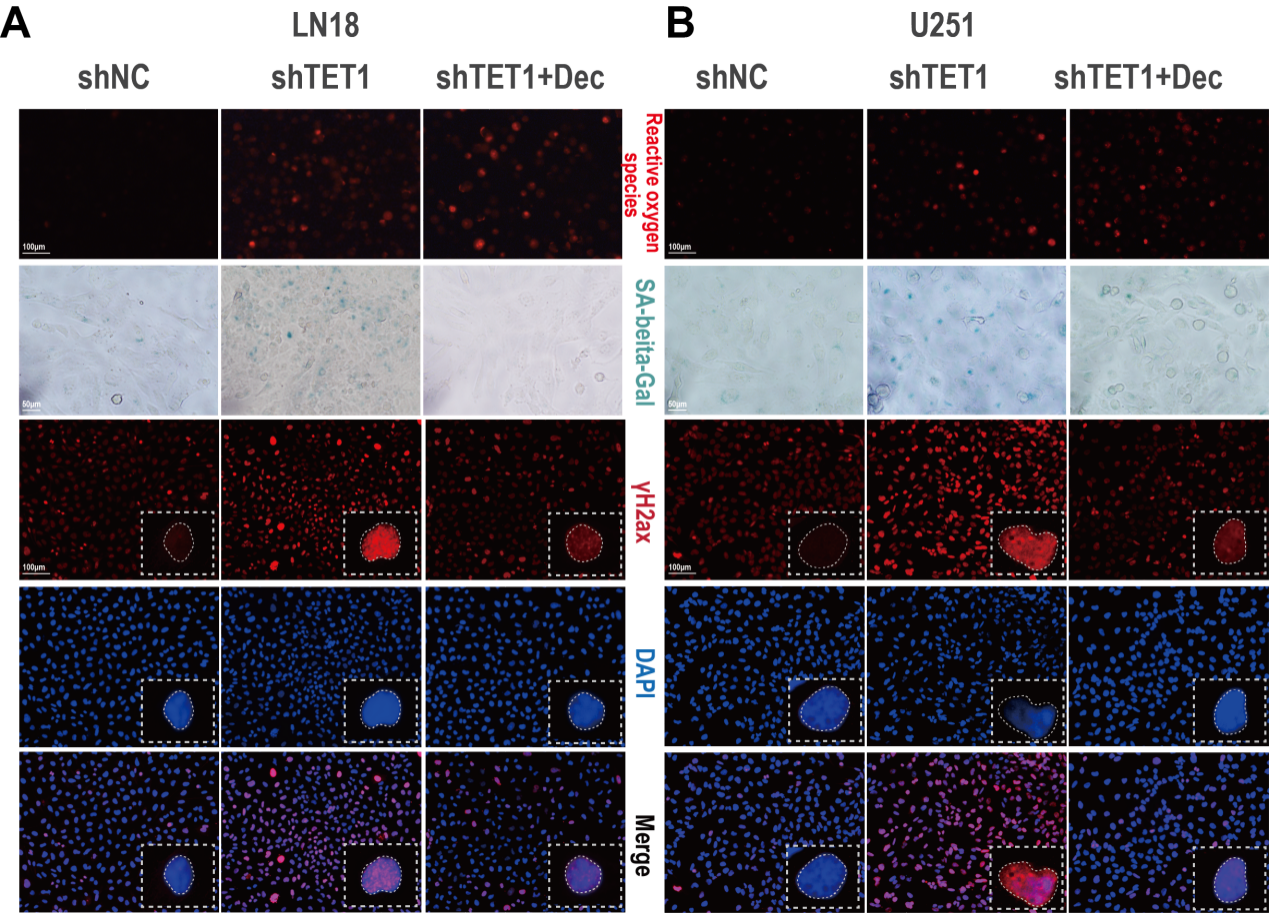


**Figure S4 TET1 modulates DNA damage and cellular senescence correlating with the regulation of methylation.** Reactive oxygen species level, SA-β-gal activity, and number of γ-H2AX foci in U251 and LN18 cells transfected with shNC, shTET1, or shTET1+decitabine (Dec).


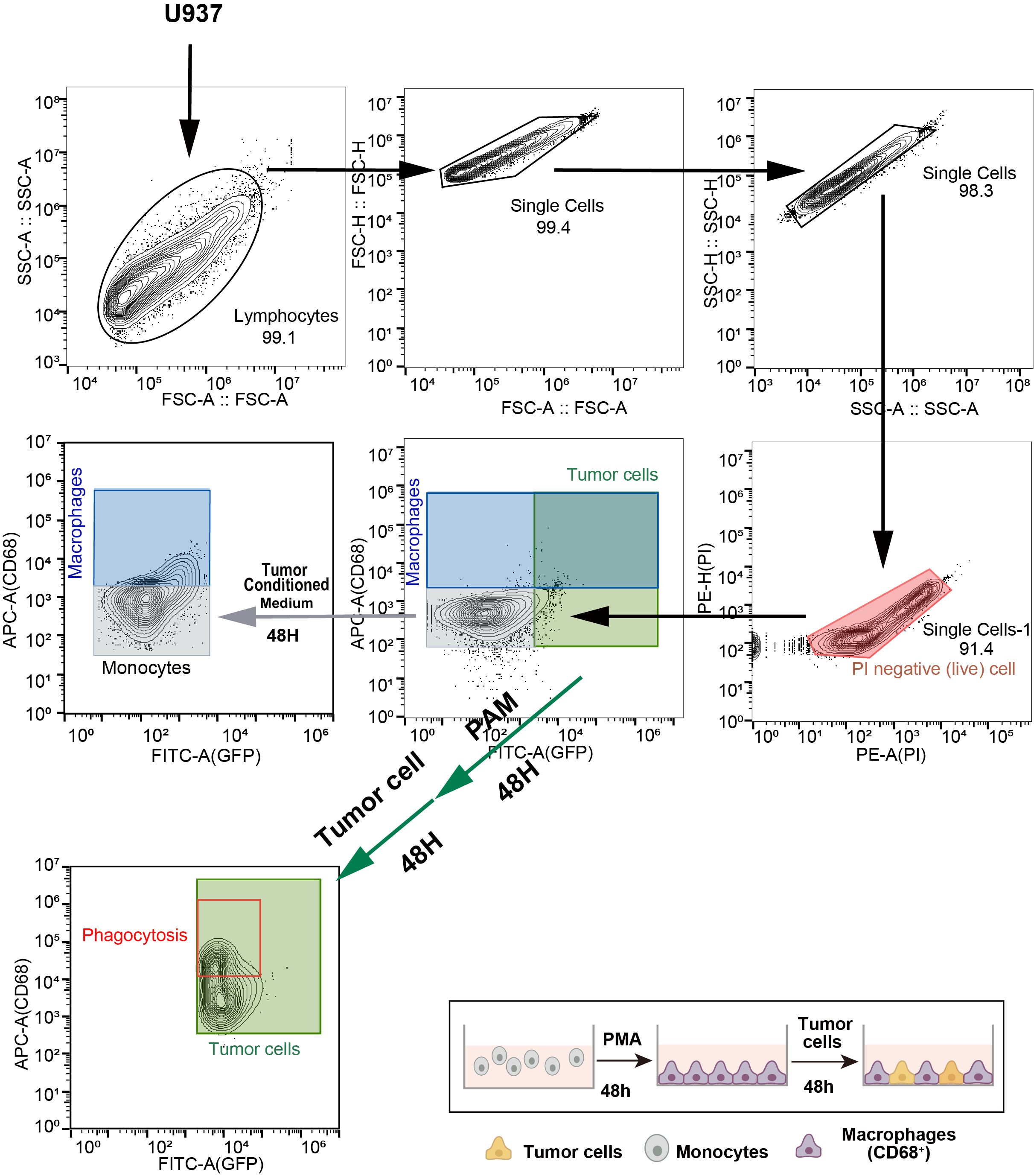


**Figure S5 Sample processing and flow cytometry gating strategy for the detection of CD68⁺GFP⁺ double-positive cells in the phagocytosis assays.**


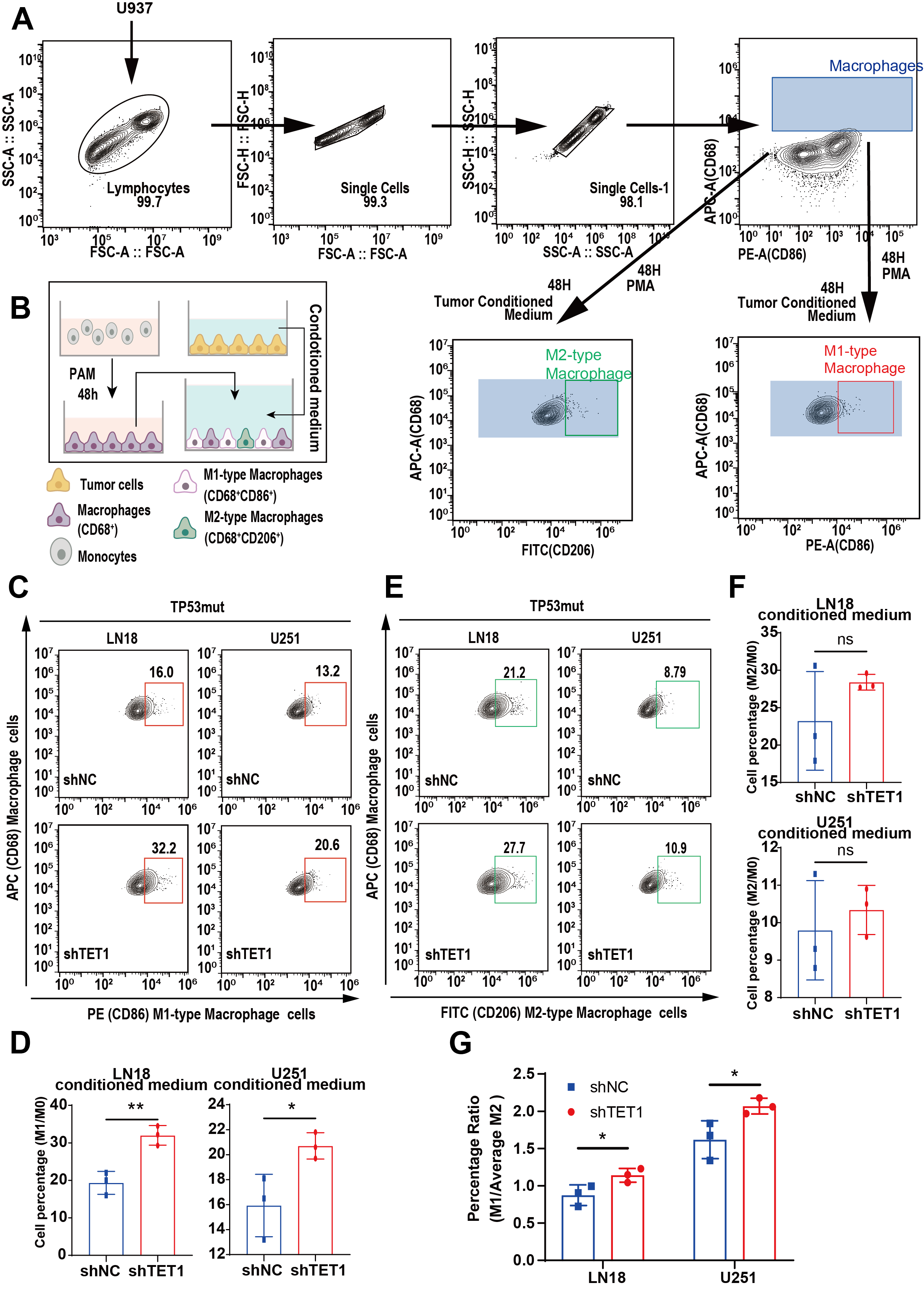


**Figure S6 TET1 knockdown promotes the polarization of M1-type TAMs in vitro.** (A) Flow cytometry gating strategy for detecting M1-type (CD68⁺CD86⁺) and M2-type (CD68⁺CD206⁺) TAMs. (B) Analysis of the role of TET1 in TAMs differentiation and polarization. (C, D) Flow cytometry analysis of the proportions of CD68^+^CD86^+^ M1-type U937-derived TAMs induced by culture with conditioned medium (CM) from U251 and LN18 cells transfected with shTET1 or shNC (C) and quantification of the percentages of CD68^+^CD86^+^ double-positive TAMs (D). (E, F) Flow cytometry analysis of the proportion of CD68^+^CD206^+^ M2-type U937-derived TAMs induced by cultured with CM from U251 and LN18 cells transfected with shTET1 or shNC (E) and quantification of the percentages of CD68^+^CD206^+^ double-positive TAMs (F). (G) Flow cytometry quantification of the ratio of M1-type/average M2-type TAMs. n=3; data are presented as the means ± SDs (D, F, and G). *P<0.05, **P<0.01.


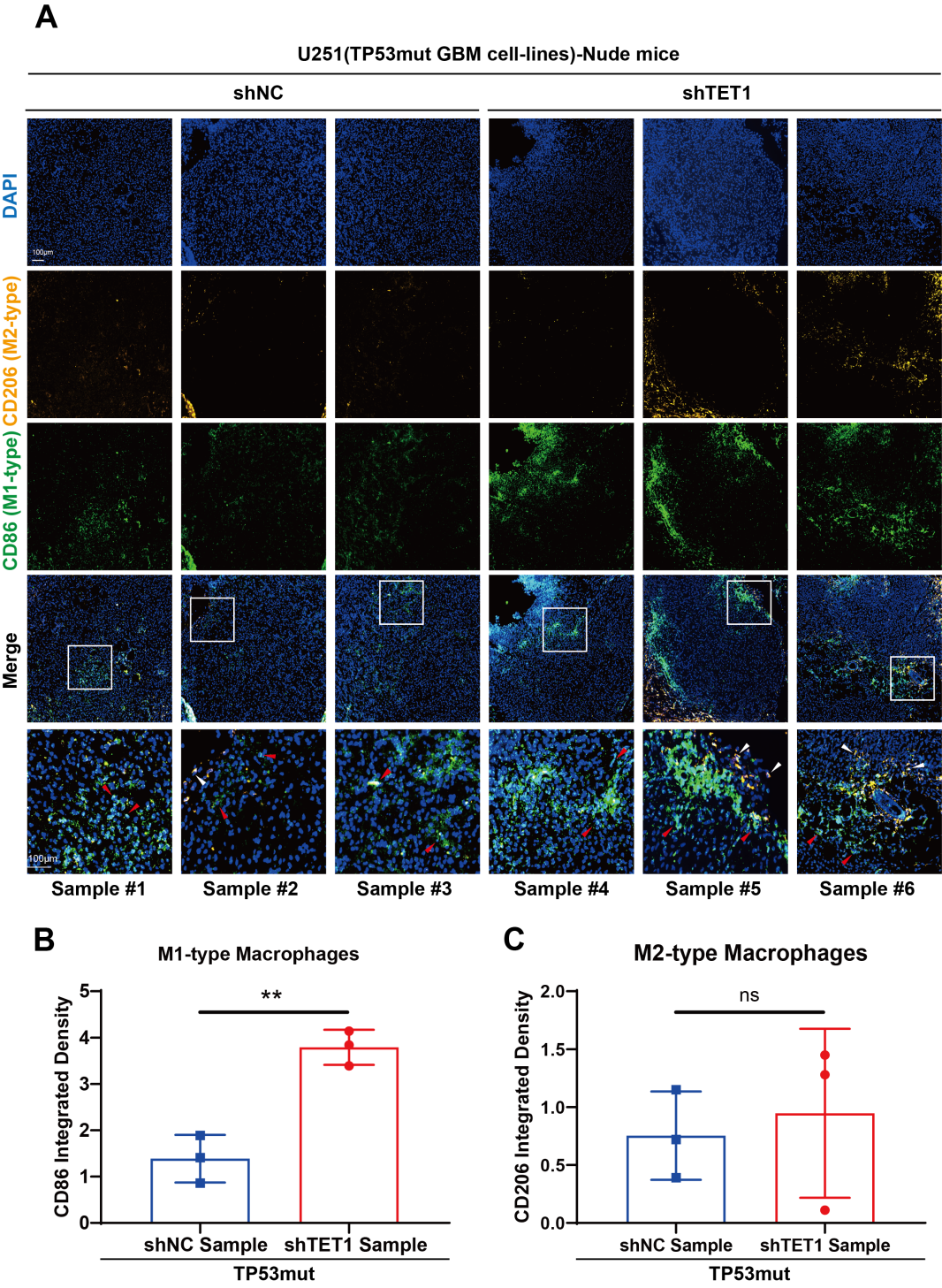


**Figure S7 TET1 knockdown promotes the infiltration and polarization of M1-type TAMs in vivo.** Immunofluorescence staining images of CD86 and CD206 in tumor sections from mice injected with U251 cells transfected with shNC or shTET1 (A) and quantification of the CD86 (B) and CD206 (C) staining intensity. CD86, green; CD206, yellow; DAPI, blue; red arrows, M1-type TAMs; white arrows, M2-type TAMs. n=3; data are presented as the means ± SDs (B, and C). **P<0.01.


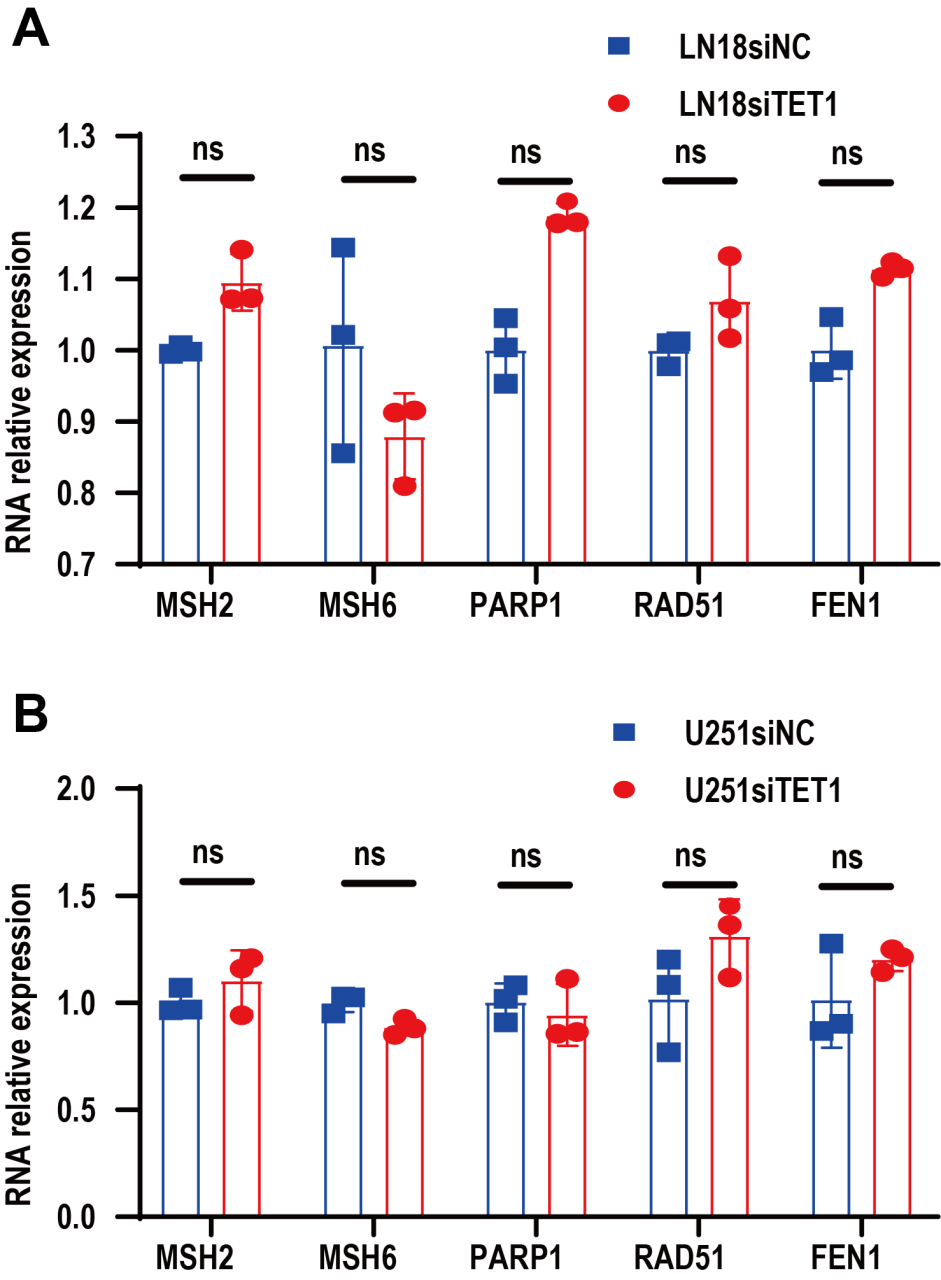


**Figure S8** TET1 knockdown dose not significantly affect the expression of DNA repair-related enzymes in U251 (A) and LN18 (B) cells. n=3; data are presented as the means ± SDs.


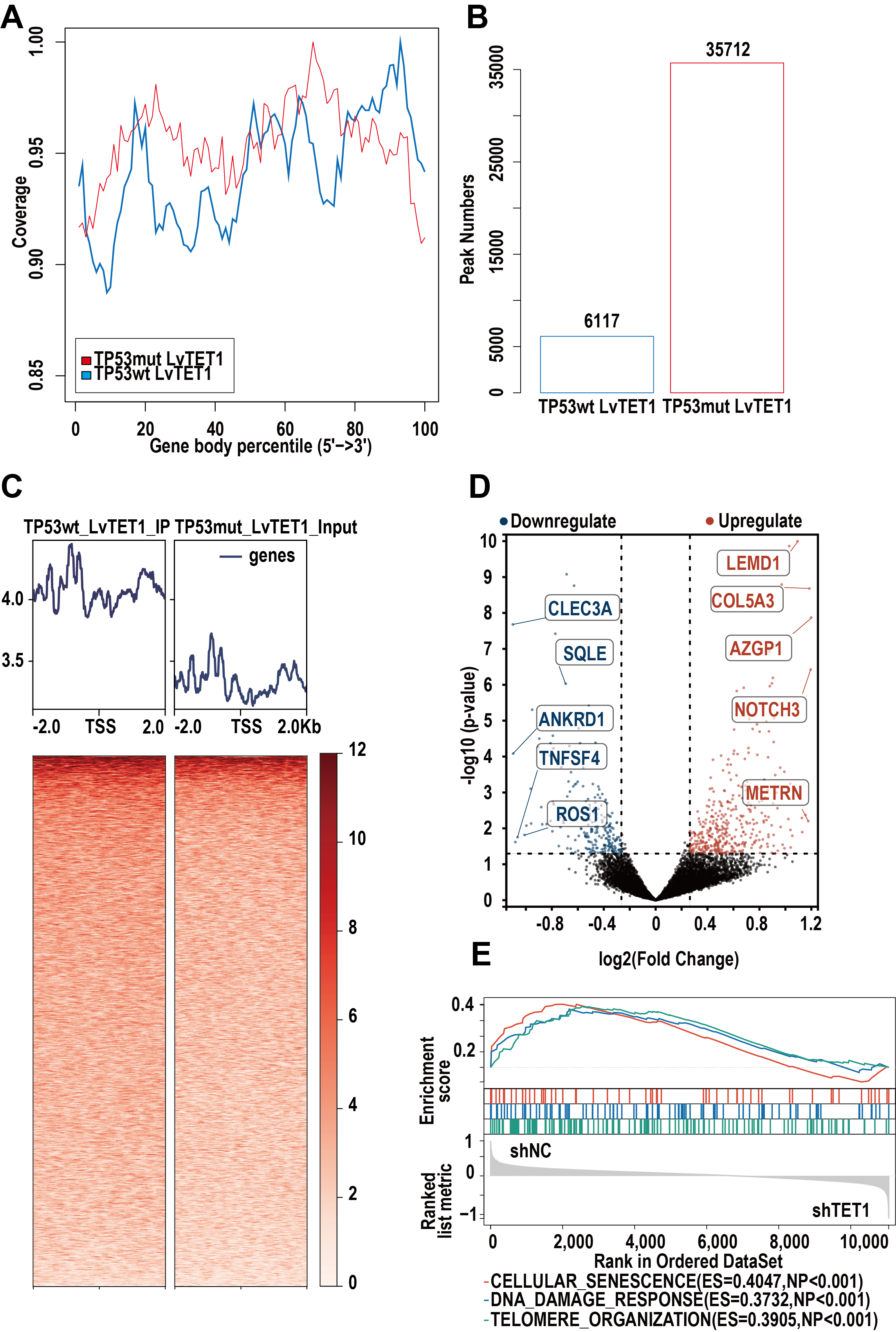


**Figure S9 Genomic modulation ability of TET1 in GBM.** (A, B) ChIP-seq profiles of TP53mut and TP53wt GBM cells with TET1 overexpression showing different peak numbers. (C) ChIP-seq profiles of TP53wt GBM cells with TET1 overexpression showing TET1 signals at the gene promoter. (D) Volcano plot of the RNA-seq data showing DEGs in U251 cells transfected with shTET1 or shNC. (E) GSEA of cellular senescence (GO 0090398), DNA damage response (GO 0030330), and telomere organization (GO 0032200) in the U251-shNC and shTET1 groups.


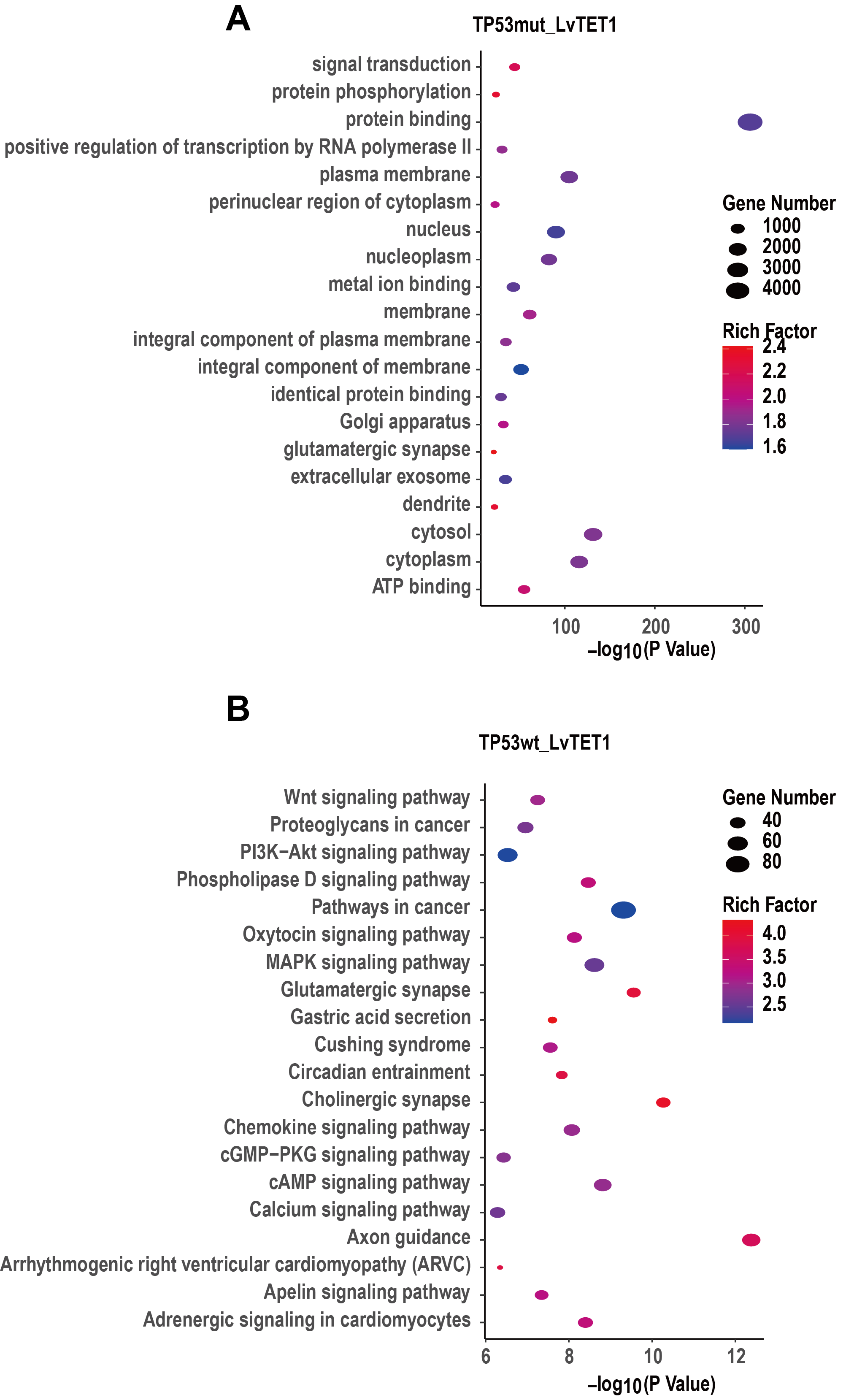


**Figure S10 Enrichment analysis of TET1-occupied genes in GBM cells.**


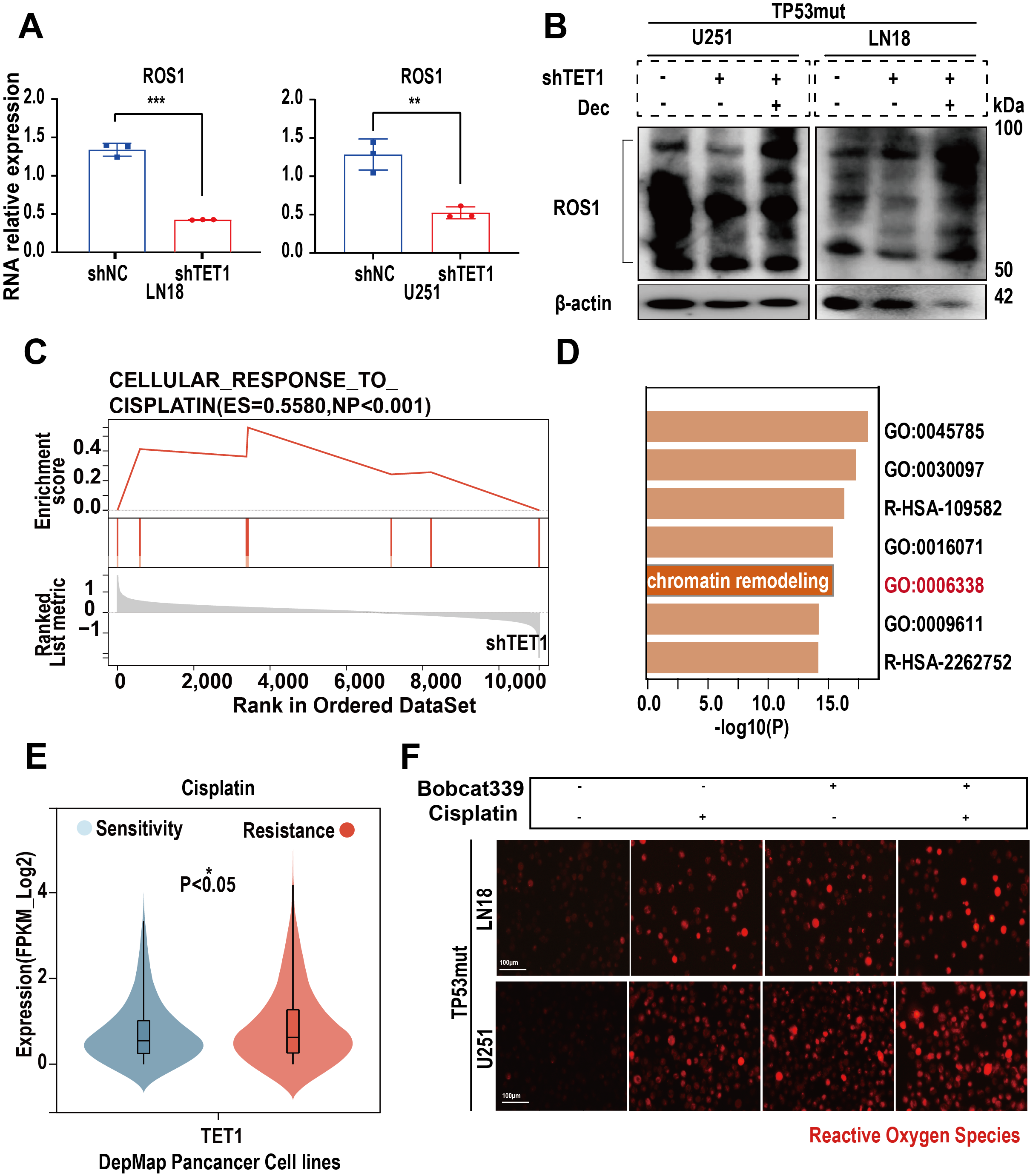


**Figure S11 TET1 regulates ROS1 expression and affects cisplatin sensitivity.** (A) ROS1 mRNA expression level in U251 and LN18 cells expressing shNC or shTET1. (B) Western blot analysis of ROS1 protein expression in U251 and LN18 cells treated with shNC, shTET1, and shTET1+Dec. (C) GSEA of the cellular response to cisplatin (GO 0072719) in U251 cells transfected with shNC or shTET1. (D) Enrichment analysis of the DEGs between cisplatin-sensitive and cisplatin-resistant pan-cancer cell lines from the DepMap database showing enrichment in chromatin remodeling pathways. (E) Expression of TET1 in cisplatin-sensitive and cisplatin-resistant pan-cancer cell lines in the DepMap database. (F) Reactive oxygen species levels in U251 and LN18 cells subjected to the indicated treatments. n=3; data are presented as the means ± SDs (A). *P<0.05, **P<0.01, ***P<0.001.


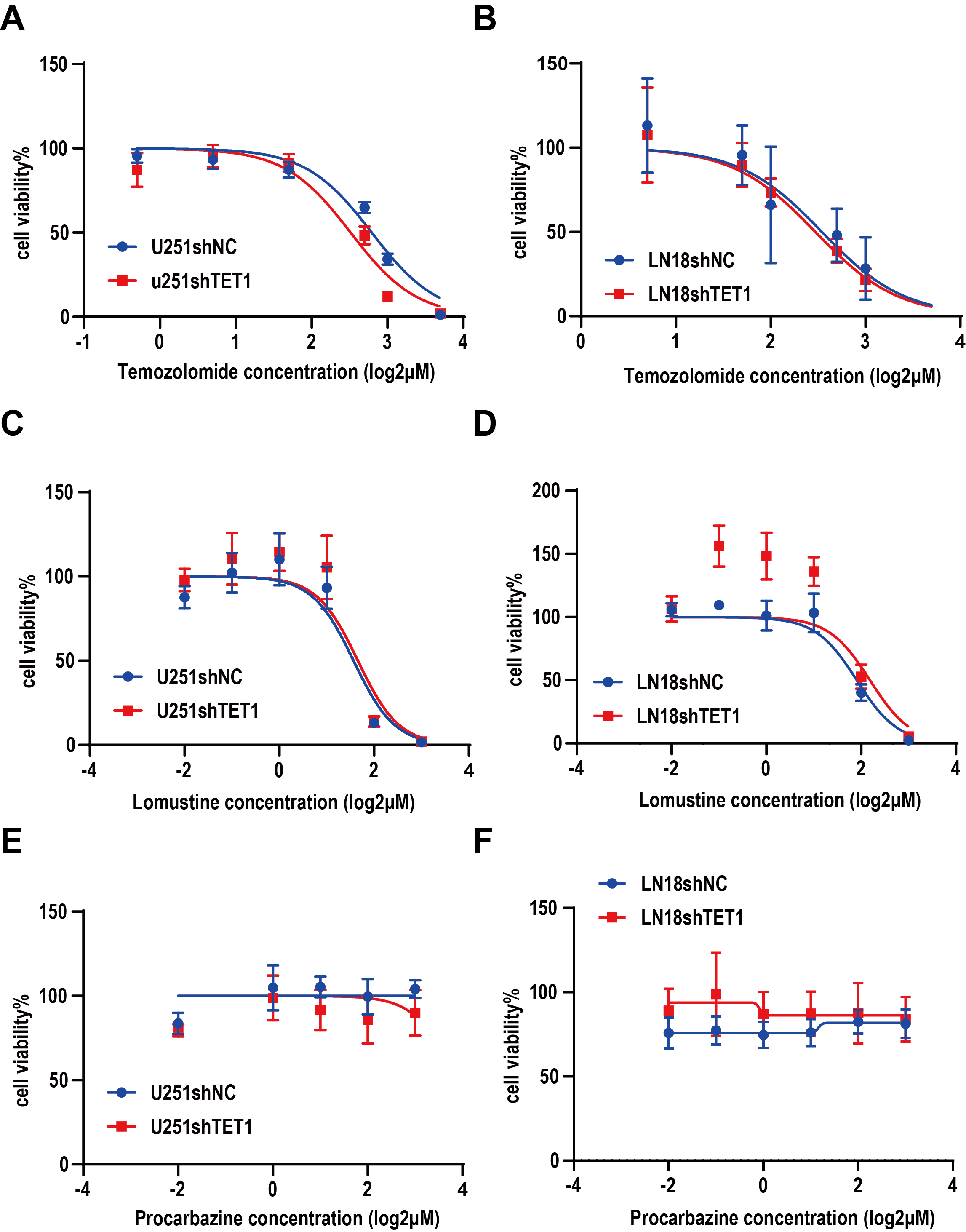


**Figure S****12 TET1 knockdown does not significantly alter sensitivity to the indicated chemotherapy agents.** (A, B) CCK-8 assay results showing the response of U251 and LN18 cells transfected with shTET1 or shNC to different doses of temozolomide. (C, D) CCK-8 assay results showing the response of U251 and LN18 cells transfected with shTET1 or shNC to different doses of lomustine. (E, F) CCK-8 assay results showing the response of U251 and LN18 cells transfected with shTET1 or shNC to different doses of procarbazine. n=3; data are presented as the means ± SDs.


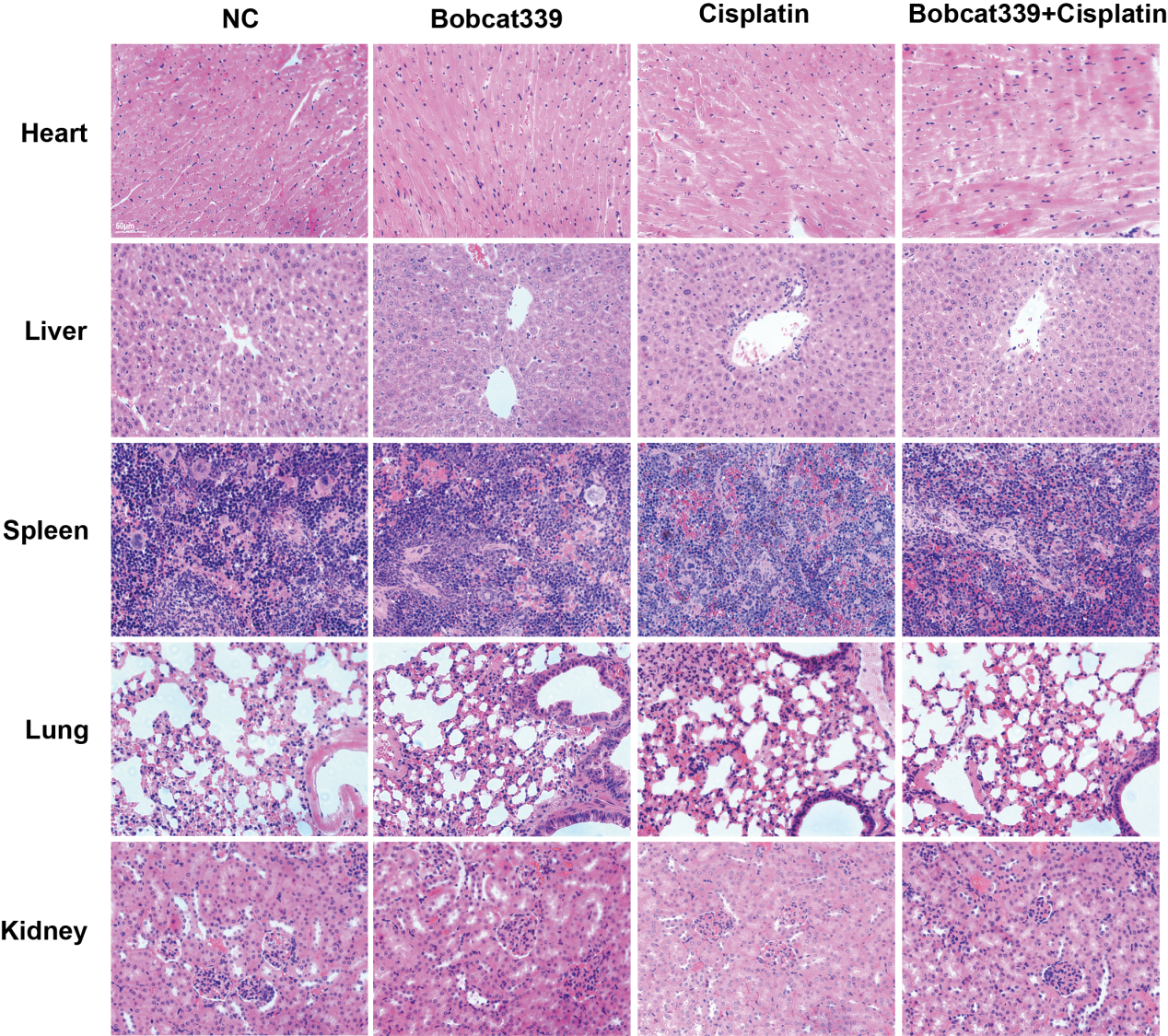


**Figure S13 Toxicity assessment by H&E staining of major organs (heart, liver, spleen, lung, and kidney) from mice on day 24 after tumor inoculation under the indicated treatment conditions.**
